# Triazole-Functionalized Jatrophone Derivatives as Antiprotozoal Agents Against *Trypanosoma cruzi*: Synthesis, Biological Evaluation and Structure—Activity Relationships

**DOI:** 10.3390/ph19050801

**Published:** 2026-05-21

**Authors:** Mariano Walter Pertino, Patricio Carreño Gonzalez, Camila Venegas González, Guillermo Schmeda-Hirschmann, Celeste Vega Gómez, Miriam Rolón, Antonieta Rojas de Arias

**Affiliations:** 1 Instituto de Química de Recursos Naturales, Universidad de Talca, Campus Lircay, Talca 3480094, Chile; p.gonzalezcarreo@gmail.com (P.C.G.); schmeda@utalca.cl (G.S.-H.); 2 Escuela de Ingeniería en Biotecnología, Facultad de Ciencias Agrarias y Forestales, Universidad Católica del Maule, Talca 3480112, Chile; camila.venegas.01@alu.ucm.cl; 3 Centro para el Desarrollo de la Investigación Científica (CEDIC), Manduvirá 635, Asunción 1255, Paraguay; mcvegagomez@gmail.com (C.V.G.); rolonmiriam@gmail.com (M.R.); rojasdearias@gmail.com (A.R.d.A.)

**Keywords:** jatrophone, click chemistry, triazole hybrids, *Trypanosoma cruzi*, antiprotozoal activity

## Abstract

**Background/Objectives**: Jatrophone is a bioactive diterpenoid with reported antitrypanosomal activity; however, its development as a lead compound is limited by pronounced cytotoxicity toward mammalian cells. This study aimed to explore the structural modification of jatrophone through triazole functionalization to modulate its antiparasitic activity and improve selectivity against *Trypanosoma cruzi*. **Methods**: A series of mono- and bis-triazole jatrophone derivatives was semi-synthesized via Cu(I)-catalyzed azide–alkyne cycloaddition (CuAAC) from a stereoselectively prepared diazido intermediate. Jatrophone, its azido precursor, and the synthesized triazole derivatives were evaluated in vitro against *T. cruzi* epimastigotes and intracellular amastigotes. Cytotoxicity toward mammalian host cells was assessed in parallel to determine selectivity indices. **Results**: Jatrophone exhibited potent activity against epimastigotes but showed poor selectivity due to significant mammalian cell toxicity. Introduction of azide and triazole functionalities altered the biological profile of the parent scaffold, leading to derivatives with reduced cytotoxicity and improved selectivity in extracellular assays. Among the evaluated compounds, a mono-triazole derivative bearing a methylene-linked cycloalkyl substituent retained antiparasitic activity while displaying markedly lower toxicity toward mammalian cells. However, in the intracellular amastigote model, most derivatives demonstrated a substantial reduction in selectivity, indicating limited translation of extracellular activity to the intracellular parasite stage. **Conclusions**: Triazole functionalization of the jatrophone scaffold represents a viable strategy to modulate its biological properties and reduce host-cell toxicity. Nevertheless, the reduced efficacy observed in intracellular assays underscores the limitations of epimastigote-based screening and highlights the challenges in developing selective intracellular antitrypanosomal agents from the jatrophone scaffold.

## 1. Introduction

Chagas disease, caused by the protozoan parasite *Trypanosoma cruzi*, affects approximately 6–7 million people worldwide and remains a major public health concern in Latin America, with increasing relevance in non-endemic regions due to migration and globalization [1,2]. Current chemotherapy relies mainly on benznidazole and nifurtimox; however, both drugs show limited efficacy in chronic infections and are frequently associated with adverse effects, highlighting the urgent need for new antitrypanosomal agents with improved safety and selectivity profiles [3,4].

The life cycle of *Trypanosoma cruzi* comprises several developmental stages that alternate between the insect vector and the mammalian host. Infective trypomastigotes enter the human body through skin or mucosal lesions and subsequently invade host cells, where they differentiate into amastigotes [5,6]. This intracellular stage is responsible for parasite replication within mammalian tissues and is closely associated with disease progression. Following host cell rupture, newly formed trypomastigotes are released, allowing the parasite to spread and infect neighboring cells [7,8].

Epimastigotes represent the replicative stage in the insect vector and are widely used for in vitro screening due to their ease of culture. However, because amastigotes constitute the clinically relevant intracellular stage, their evaluation is essential for identifying compounds with true therapeutic potential. Consequently, assessing antiparasitic activity against both epimastigote and amastigote forms provides a more comprehensive and biologically meaningful evaluation of new antichagasic agents [9,10].

Natural products continue to play a central role in antiparasitic drug discovery by providing structurally complex and biologically validated scaffolds [11]. Among them, the diterpene jatrophone, the major terpenoid constituent of the rhizomes of *Jatropha isabelli* Muell. Arg., has attracted considerable attention since its first isolation as an antileukemic agent from *Jatropha gossypiifolia* [12,13]. Subsequent studies revealed a broad spectrum of biological activities, including antiproliferative, antiprotozoal, and enzyme-modulating effects [14,15,16,17].

Jatrophone displays significant in vitro and in vivo activity against *Leishmania* spp. and *T. cruzi*. Nevertheless, its pronounced cytotoxicity severely limits its therapeutic potential [17]. This unfavorable profile has been attributed, at least in part, to the presence of multiple electrophilic centers within its structure, particularly the α,β-unsaturated carbonyl system capable of undergoing Michael-type addition with biological thiols [18]. Such nonspecific reactivity has been proposed as a key contributor to its cytotoxic effects.

**Scheme 1:**
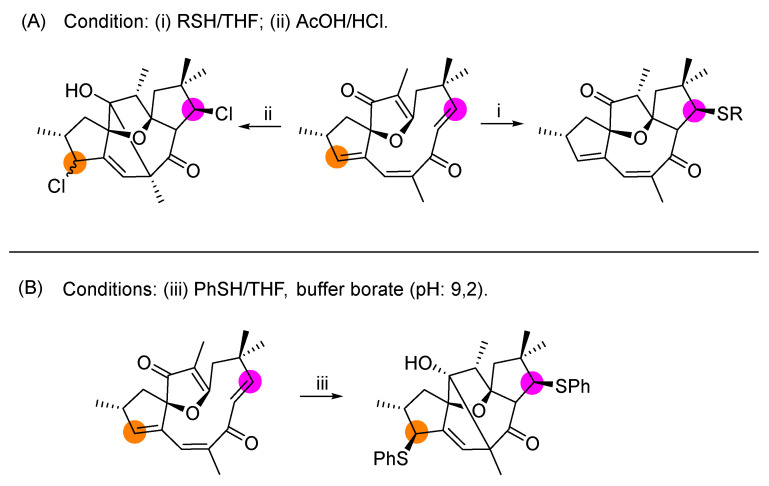
(**A**) Synthesis of some Jatrophone derivates by Kupchan et al. [12,13,19]. (**B**) Jatrophone transformation by the Hady group [20]. Highlighted colored circles indicate the reactive positions involved in the structural modifications.

Despite its biological relevance, relatively few structural modifications of jatrophone have been reported. Early studies by Kupchan and co-workers focused on antitumor-oriented derivatization (Scheme 1A) [12,13,19], while more recent work by Hady et al. [20], explored nucleophilic additions of thiols to the jatrophone scaffold (Scheme 1B), yielding derivatives with altered biological profiles and, in some cases, reduced cytotoxicity. Importantly, these studies suggest that modulation of the electrophilic character of jatrophone can attenuate toxicity while retaining antiparasitic activity.

1,2,3-Triazoles are widely recognized as privileged pharmacophores in medicinal chemistry due to their remarkable chemical and metabolic stability, high dipole moment, and ability to function as hydrogen-bond acceptors as well as rigid amide bioisosteres [21,22]. These features enable favorable interactions with biological targets while often improving aqueous solubility, binding affinity, and pharmacokinetic properties [23,24]. In antiparasitic drug discovery, triazole-containing derivatives have demonstrated significant activity against *Trypanosoma cruzi*, *Leishmania* spp., and other protozoan pathogens, frequently exhibiting enhanced selectivity and reduced toxicity compared to their parent scaffolds [25,26,27,28]. Beyond serving as a simple linker, the triazole ring can actively contribute to molecular recognition through π–π stacking, dipole–dipole interactions, and coordination with amino acid residues at target binding sites [24,29]. Based on these considerations, we hypothesized that the incorporation of triazole units into the jatrophone scaffold could expand its chemical space, modulate the electrophilic character of the parent diterpene, and potentially improve its antiparasitic selectivity profile.

Herein, we report the synthesis of a series of novel jatrophone–triazole derivatives obtained through azidation followed by Cu(I)-catalyzed azide–alkyne cycloaddition, together with their evaluation against *T. cruzi* epimastigotes and intracellular amastigotes. A detailed structure–activity relationship (SAR) analysis is presented, providing insight into the impact of triazole functionalization on antiparasitic activity and mammalian cytotoxicity.

## 2. Results and Discussion

### 2.1. Chemistry

**Scheme 2:**
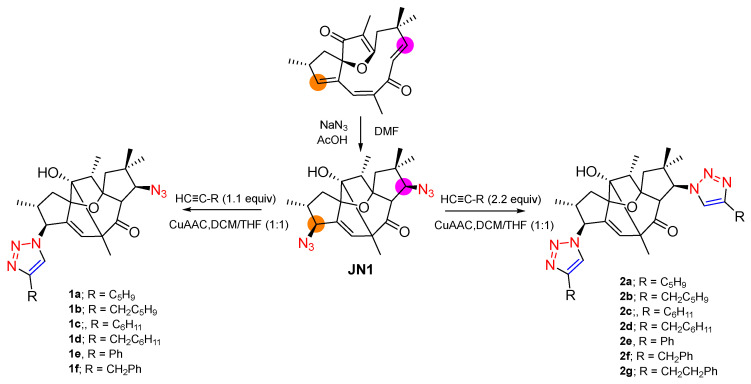
Synthesis of Jatrophone-triazole derivates.

Jatrophone was used as the starting material for the synthesis of triazole-functionalized derivatives. Treatment of jatrophone with sodium azide afforded the diazido intermediate **JN1** in good yield and with high stereoselectivity (Scheme S1, Supplementary Materials). Subsequent Cu(I)-catalyzed azide–alkyne cycloaddition (CuAAC) with a series of terminal alkynes yielded two families of derivatives: mono-triazole compounds (**1a**–**1f**), obtained predominantly using 1.1 equivalents of alkyne, and bis-triazole compounds (**2a**–**2g**), formed preferentially when 2.2 equivalents of alkyne were employed relative to **JN1** (Scheme 2). Under the mono-functionalization conditions, trace amounts of bis-triazole derivatives were also detected by TLC and crude NMR analysis, whereas increasing the alkyne loading and/or reaction time favored formation of the bis-substituted products. No evidence for alternative mono-triazole regioisomers arising from cycloaddition at the more hindered azide position was observed.

Comparison of the 2D NMR data of mono-triazole derivative **1d** with those of the diazido precursor **JN1** supported selective mono-functionalization (Tables S1–S3). In particular, only one azide-bearing methine environment underwent a significant chemical shift change after cycloaddition, while HMBC, HSQC, COSY, and NOESY experiments confirmed the proposed structural assignment. Visual inspection of a minimized three-dimensional model suggested that the second azide group is located in a sterically congested region close to a gem-dimethyl-substituted carbon center, likely hindering approach of the copper–acetylide species during the cycloaddition process. Preliminary Gasteiger charge analysis revealed very similar charge distributions for both azide groups, suggesting that steric accessibility and conformational constraints, rather than electronic effects, govern the observed regioselectivity. Detailed 2D NMR correlations and NOESY analyses are provided in the Supplementary Materials.

All compounds were characterized by ^1^H and ^13^C NMR spectroscopy, including two-dimensional NMR experiments, as well as high-resolution mass spectrometry (HRMS) (Figures S1–S51). Compound purity was assessed by ^1^H NMR spectroscopy, showing >95% purity based on signal integration.

### 2.2. Biological Evaluation Strategy and Experimental Models

The antitrypanosomal activity of jatrophone (**JAT**), its azido intermediate (**JN1**), and the corresponding triazole derivatives was evaluated using a tiered in vitro screening strategy. Two complementary *Trypanosoma cruzi* models were employed: CL Brener epimastigotes, representing the replicative stage in the insect vector, and Tulahuen β-galactosidase-expressing intracellular amastigotes, which constitute the clinically relevant intracellular form of the parasite.

Initial screening against epimastigotes was conducted to identify active compounds and establish preliminary structure–activity relationships (SAR) (Table 1). Compounds displaying measurable antiparasitic activity were subsequently evaluated against intracellular amastigotes to assess their efficacy in a biologically relevant context (Table 2). In parallel, cytotoxicity assays were performed using murine L929 fibroblasts and Vero cells to determine host cell toxicity and calculate selectivity indices (SI), as summarized in Table 1 and Table 2.

#### 2.2.1. Activity Against *T. cruzi* Epimastigotes: SAR and Selectivity Trends

Jatrophone (**JAT**) displayed high potency against epimastigotes (IC_50_ = 1.7 µM), but this activity was accompanied by pronounced cytotoxicity toward L929 fibroblasts, resulting in a poor selectivity index (SI = 3.3). This behavior is consistent with previous reports describing jatrophone as a highly reactive diterpenoid whose biological activity is limited by nonspecific toxicity [17,30].

Notably, conversion of JAT into the azido derivative **JN1** resulted in a moderate decrease in antiparasitic potency (IC_50_ = 4.1 µM) but a marked reduction in cytotoxicity (IC_50_ = 116.9 µM), leading to a substantially improved SI (28.5). This result supports our initial hypothesis that partial modulation of the electrophilic character of the jatrophone scaffold can reduce toxicity while preserving biological activity, in agreement with earlier derivatization studies reported for jatrophone [12,13,20].

The triazole derivatives revealed clear SAR trends. In series 1 (**1a**–**1f**), compounds in which a cycloalkyl (**1a**, **1c**) or aryl (**1e**) moiety is directly attached to the triazole ring showed moderate antiparasitic activity but poor selectivity (SI ≤ 2.2), indicating limited discrimination between parasite and mammalian cells. In contrast, analogues in which the same cyclic moiety is connected through an additional methylene spacer (–CH_2_–) (**1b**, **1d**, **1f**) displayed improved safety profiles, suggesting that spatial separation between the triazole core and the hydrophobic ring plays a critical role in modulating biological selectivity. Among these compounds, **1b** emerged as the most promising derivative, combining good antiparasitic potency (IC_50_ = 4.8 µM) with no detectable cytotoxicity up to 256 µM, resulting in an outstanding selectivity index (SI ≥ 53). Compound **1f** also benefited from this structural modification, maintaining moderate activity alongside low cytotoxicity (SI ≥ 11.2). However, compound **1d**, despite sharing the same methylene-linked architecture, showed a pronounced loss of antiparasitic potency (IC_50_ = 127 µM), highlighting that the presence of the spacer alone is not sufficient to preserve activity and that subtle differences in ring nature and spatial orientation critically influence target engagement.

In series 2 (**2a**–**2g**), in which two triazole units are linked to the jatrophone core, all derivatives exhibited moderate and relatively homogeneous activity against *T. cruzi* epimastigotes (IC_50_ ≈ 20–36 µM), with no evident correlation between antiparasitic potency and the chemical nature of the triazole substituents. In contrast, marked differences emerged in mammalian cytotoxicity, indicating that selectivity in this series is primarily governed by host-cell tolerance rather than intrinsic anti-parasitic activity, in line with phenotypic screening approaches in Chagas disease drug discovery where antiparasitic effects and host-cell toxicity are evaluated in parallel to identify selective compounds [31,32].

Compounds bearing short aliphatic substituents directly attached to the triazole rings (**2a**–**2c**) showed limited selectivity (SI ≤ 2.6), in agreement with the trend observed in series 1, except for the derivative **1b**, which exhibited an improved selectivity profile. Notably, derivatives **2d**–**2g** were non-cytotoxic toward L929 fibroblasts up to 256 µM, resulting in higher selectivity indices (SI ≥ 9.6–11.1) without significant loss of antiparasitic potency. In this series, derivatives incorporating a spacer between the triazole ring and the hydrophobic moiety tended to display reduced cytotoxicity, as illustrated by compound **2g** bearing a –CH_2_–CH_2_–phenyl substituent. Overall, these results are consistent with the SAR trends observed in series 1, highlighting the influence of the linkage mode to the triazole scaffold on biological selectivity.

#### 2.2.2. Activity Against Intracellular Amastigotes: Pharmacological Relevance and Limitations

Given that intracellular amastigotes represent the clinically relevant stage of *T. cruzi*, selected compounds showing favorable profiles against epimastigotes were further evaluated using the Tulahuen β-galactosidase amastigote assay in Vero cells. In contrast to the epimastigote data, most compounds displayed a general loss of selectivity in this more demanding intracellular model.

The azido derivative **JN1**, despite its excellent selectivity index against epimastigotes, exhibited marked cytotoxicity toward Vero host cells (IC_50_ = 0.7 µM), resulting in a very low selectivity index (SI = 0.5) in the amastigote assay. This discrepancy illustrates a well-recognized limitation of extracellular screening models, in which improvements in apparent selectivity do not necessarily translate to intracellular or host-cell–based systems. Similar observations have been reported in *T. cruzi* drug discovery studies, where both compound activity and host-cell susceptibility vary significantly depending on the cellular model employed [33]. In this context, assays evaluating both intracellular antiparasitic activity and host-cell viability provide a more realistic assessment of compound selectivity, as potent parasite inhibition may still be accompanied by substantial host-cell toxicity. For **JN1**, the results obtained in this assay indicate that its antiparasitic effect is largely offset in a host-cell environment, possibly due to increased cellular exposure or residual nonspecific effects.

Among the triazole derivatives, compounds **1b**, **1f**, **2d**, **2e**, and **2g** retained measurable activity against intracellular amastigotes; however, their selectivity indices remained modest (SI = 0.4–1.6). Within this group, compound **2d** emerged as the most balanced derivative (IC_50_ = 3.5 µM; SI = 1.6), although overall selectivity across the series remained limited. In contrast, benznidazole exhibited excellent selectivity in the amastigote model (SI > 80), reflecting its long-standing use as a reference drug in intracellular *T. cruzi* assays and the demanding criteria applied in Chagas drug discovery [34,35].

Overall, the divergence between epimastigote and amastigote data observed for the jatrophone-derived series aligns with previous reports showing that compounds identified as hits in epimastigote-based screenings frequently lose selectivity in intracellular amastigote assays. This outcome reflects the greater biological complexity of intracellular infection models, in which factors such as host-cell permeability, intracellular accumulation, and host toxicity often become the primary constraints on translational potential [36].

Although the precise molecular targets of the synthesized derivatives remain unknown, several mechanisms have been proposed for structurally related terpenoids and triazole-containing compounds in *Trypanosoma cruzi*, including disruption of mitochondrial function, oxidative stress induction, membrane perturbation, and inhibition of sterol biosynthesis pathways, particularly CYP51 [37,38,39,40]. Furthermore, triazole derivatives have emerged as privileged scaffolds in antiparasitic drug discovery due to their ability to interact with essential parasite enzymes and metabolic pathways [38,41,42]. However, no target engagement or mechanistic studies were performed in the present work; therefore, the observed antiparasitic activity should currently be considered phenotypic [37]. Future investigations will focus on elucidating the molecular targets and mechanism of action of the most promising derivatives.

### 2.3. In Silico ADME Profile and Relationship with Biological Activity

The physicochemical and pharmacokinetic properties of **JN1** and the triazole derivatives were predicted using SwissADME web tool [43] to rationalize the observed biological trends (Table S4, Supplementary Materials). Overall, triazole functionalization increased molecular weight (≈400–660 g/mol) and lipophilicity (Log P ≈ 2.4–5.4), while maintaining relatively high polarity (TPSA ≈ 108–146 Å^2^) and low predicted gastrointestinal absorption across the series.

**JN1** displayed the highest polarity (TPSA = 146.03 Å^2^) and moderate lipophilicity (Log P = 2.44), consistent with its improved selectivity in the epimastigote model compared to jatrophone. However, its poor performance in the intracellular assay suggests that favorable selectivity in L929 cells is not predictive of behavior in host-cell infection models.

Within the mono-triazole series (**1a**–**1f**), compounds showed similar ADME profiles (Log P ≈ 3.3–3.6; TPSA ≈ 127 Å^2^), indicating that differences in biological activity are not primarily driven by global physicochemical parameters. Notably, compound **1b**, the most selective derivative, does not significantly differ in calculated properties from less selective analogues, highlighting the importance of structural features such as the methylene spacer rather than bulk ADME descriptors.

The bis-triazole derivatives (**2a**–**2g**) exhibited higher lipophilicity (Log P up to ~5.3) and lower polarity (TPSA ≈ 108 Å^2^), yet maintained moderate antiparasitic activity. In this series, selectivity was mainly associated with reduced cytotoxicity rather than enhanced potency, particularly for compounds **2d**–**2g**.

A consistent trend across both series is that the introduction of a spacer between the triazole ring and the hydrophobic substituent correlates with improved selectivity. While this effect is primarily structural, the resulting balance between lipophilicity and polarity may contribute to reduced mammalian toxicity. Despite these favorable trends in the epimastigote model, most compounds showed limited selectivity in the intracellular amastigote assay. This suggests that, although their physicochemical properties are compatible with cell permeability, host-cell toxicity remains a major limitation.

Overall, the ADME analysis supports that triazole incorporation effectively modulates the physicochemical profile of the jatrophone scaffold but also highlights that improvements in extracellular selectivity do not necessarily translate to intracellular efficacy, emphasizing the need for further optimization toward reduced host-cell toxicity.

## 3. Materials and Methods

### 3.1. General Procedures

^1^H and ^13^C NMR spectra were recorded at 400 MHz for ^1^H and 100 MHz for ^13^C using a Bruker Avance 400 spectrometer (Bruker Corporation, Rheinstetten, Germany), except for one compound, which was recorded at 500 MHz for ^1^H and 125 MHz for ^13^C on a Bruker Avance III 500 spectrometer (Bruker Corporation, Rheinstetten, Germany). Chemical shifts (δ) are reported in parts per million (ppm) relative to residual solvent signals and coupling constants (*J*) are reported in Hertz (Hz). High-resolution mass spectra (HRMS) were acquired by direct infusion using an electrospray ionization (ESI) source in positive ion mode on a Bruker compact™ QTOF mass spectrometer (Bruker Corporation, Rheinstetten, Germany) at the Pontificia Universidad Católica de Chile. Flash column chromatography was carried out using silica gel 60 (230–400 mesh) from Merck KGaA, Darmstadt, Hesse, Germany. Analytical thin layer chromatography (TLC) was performed using silica gel on aluminum sheets (Merck KGaA, Darmstadt, Hesse, Germany).

### 3.2. Plant Material

Jatrophone used for the preparation of the derivatives was isolated from the rhizomes of *Jatropha isabelli*, as previously described by our group [44].

### 3.3. Synthesis of the Compounds

#### 3.3.1. Synthesis of Azido-Jatrophone (**JN1**)

To a stirred solution of jatrophone (1.0 equiv.) in dry N,N-dimethylformamide (DMF, 1 mL) at room temperature was added sodium azide (NaN_3_, 5.0 equiv.) followed by a few drops of acetic acid to promote the reaction. The reaction mixture was stirred at room temperature for 20 h, and progress was monitored by TLC. Upon completion, the reaction was quenched by addition of water and extracted with ethyl acetate (EtOAc). The combined organic layers were dried over anhydrous Na_2_SO_4_, filtered, and concentrated under reduced pressure. The crude product was purified by silica gel column chromatography (n-hexane/EtOAc 7:3) to afford azido-jatrophone (**JN1**) in 54% as a white solid. R_f_ = 0.86 (n-hexane/EtOAc 4:6). ^1^H NMR (400 MHz, CDCl_3_) δ 5.60 (s, 1H), 3.70 (d, *J* = 5.5 Hz, 1H), 3.68 (d, *J* = 10.9 Hz, 1H), 3.25 (d, *J* = 11.0 Hz, 1H), 2.64 (m, 1H), 2.00 (q, *J* = 7.3 Hz, 1H), 1.91–1.86 (m, 2H), 1.74 (d, *J* = 15.0 Hz, 1H), 1.57 (dd, *J* = 13.6, 9.7 Hz, 1H), 1.34 (s, 3H), 1.09 (d, *J* = 5.3 Hz, 3H), 1.08 (s, 3H), 1.01 (s, 3H) 0.86 (d, *J* = 7.3 Hz, 3H). ^13^C NMR (101 MHz, CDCl_3_) δ 210.67, 150.96, 135.31, 101.22, 90.28, 86.35, 70.94, 70.48, 64.57, 57.29, 48.69, 45.47, 43.15, 40.57, 38.25, 27.23, 22.88, 18.68, 14.59, 8.04. HRMS (ESI) *m*/*z*: calcd for C_20_H_26_N_6_O_3_ [M + NH_4_]^+^ 416.2404, found 416.2410.

#### 3.3.2. General CuAAC Procedure for Jatrophone–Triazole Derivatives

A solution of **JN1** (1.0 equiv.) in dichloromethane (DCM) and tetrahydrofuran (THF) mixed 1:1 (2 mL total) was treated with the corresponding terminal alkyne and catalytic amounts of CuSO_4_·5H_2_O (20 mol%) and sodium ascorbate (40 mol%) to generate Cu(I) in situ. The alkyne was added in either 1.1 equivalents (for derivatives **1a**–**1f**) or 2.2 equivalents (for derivatives **2a**–**2g**) relative to **JN1**. The reaction mixture was stirred at room temperature until consumption of the azide was observed by TLC.

After reaction completion, the mixture was diluted with DCM (20 mL) and washed successively with water and brine. The organic phase was separated, dried over Na_2_SO_4_, filtered, and concentrated under reduced pressure. The crude residue was purified by silica gel column chromatography (eluent gradients depending on product polarity) to give the respective jatrophone–triazole derivatives in good to moderate yields. Systematic IUPAC names and SMILES representations of all synthesized compounds are provided in Table S5 (Supplementary Materials).

#### 3.3.3. Characterization of the Products

Compound **1a**. **JN1** (40 mg, 0.10 mmol) and cyclopentylacetylene (13 μL, 0.11 mmol) were reacted according to the general procedure. Purification by flash column chromatography (n-hexane/EtOAc 6:4) afforded **1a** (30 mg, 61%) as a white solid. R_f_ = 0.58 (n-hexane/EtOAc 4:6). ^1^H NMR (400 MHz, CDCl_3_) δ 7.47 (s, 1H), 5.47 (s, 1H), 5.17 (d, *J* = 7.2 Hz, 1H), 4.52 (br s, 1H, OH), 3.37 (d, *J* = 11.0 Hz, 1H), 3.26 (d, *J* = 11.0 Hz, 1H), 3.11 (m, 1H), 2.77 (m, 1H), 2.16–2.01 (m, 3H), 1.96–1.85 (m, 2H), 1.83–1.76 (m, 2H), 1.74–1.65 (m, 2H), 1.64–1.57 (m, 2H), 1.55–1.49 (m, 2H), 1.30 (s, 3H), 1.18 (d, *J* = 6.8 Hz, 3H), 1.08 (s, 3H), 1.03 (s, 3H), 0.86 (d, *J* = 7.3 Hz, 3H). ^13^C NMR (101 MHz, CDCl_3_) δ 210.05, 153.71, 150.01, 135.49, 117.53, 102.30, 90.21, 86.91, 71.01, 70.78, 64.04, 57.83, 48.31, 47.16, 45.46, 40.66, 38.69, 36.79, 33.25, 33.23, 27.03, 25.24, 25.22, 23.20, 18.42, 14.56, 8.14. HRMS (ESI) *m*/*z*: calcd for C_27_H_36_N_6_O_3_ [M + H]^+^ 493.2922, found 493.2938.

Compound **1b**. **JN1** (40 mg, 0.10 mmol) and 3-cyclopentyl-1-propyne (14 μL, 0.11 mmol) were reacted according to the general procedure. Purification by flash column chromatography (n-hexane/EtOAc 6:4) afforded **1b** (29 mg, 58%) as a white solid. R_f_ = 0.53 (n-hexane/EtOAc 4:6). ^1^H NMR (400 MHz, CDCl_3_) δ 7.50 (s, 1H), 5.50 (s, 1H), 5.17 (d, *J* = 7.3 Hz, 1H), 3.77 (br s, 1H, OH), 3.40 (d, *J* = 11.0 Hz, 1H), 3.29 (d, *J* = 11.0 Hz, 1H), 2.86 (m, 1H), 2.69 (d, *J* = 7.3 Hz, 2H), 2.18–2.05 (m, 2H), 1.95–1.91 (m, 2H), 1.83–1.77 (m, 2H), 1.75–1.69 (m, 2H), 1.65–1.55 (m, 2H), 1.54– 1.48 (m, 2H), 1.33 (s, 3H), 1.23 (d, *J* = 6.7 Hz, 3H), 1.19–1.14 (m, 2H), 1.11 (s, 3H), 1.05 (s, 3H), 0.89 (d, *J* = 7.2 Hz, 3H). ^13^C NMR (101 MHz, CDCl_3_) δ 209.84, 150.22, 148.73, 135.32, 118.88, 102.11, 90.12, 86.90, 70.91, 70.78, 63.88, 57.92, 48.38, 46.97, 45.49, 40.64, 39.73, 38.69, 32.61 (2C), 31.82, 27.12, 25.04, 25.01, 23.15, 18.38, 14.50, 8.09. HRMS (ESI) *m*/*z*: calcd for C_28_H_38_N_6_O_3_ [M + H]^+^ 507.3078, found 507.3085.

Compound **1c**. **JN1** (40 mg, 0.10 mmol) and cyclohexylacetylene (14 μL, 0.11 mmol) were reacted according to the general procedure. Purification by flash column chromatography (n-hexane/EtOAc 6:4) afforded **1c** (26 mg, 51%) as a white solid. R_f_ = 0.58 (n-hexane/EtOAc 4:6). ^1^H NMR (400 MHz, CDCl_3_) δ 7.45 (s, 1H), 5.45 (s, 1H), 5.10 (d, *J* = 7.0 Hz, 1H), 4.25 (br s, 1H, OH), 3.35 (d, *J* = 11.0 Hz, 1H), 3.24 (d, *J* = 11.0 Hz, 1H), 2.76 (m, 1H), 2.72–2.61 (m, 1H), 2.08 (m, 1H), 2.01–1.95 (m, 2H), 1.90–1.86 (m, 2H), 1.78–1.75 (m, 2H), 1.74–1.70 (m, 2H), 1.68–1.64 (m, 1H), 1.37–1.29 (m, 4H), 1.29 (s, 3H), 1.22–1.20 (m, 1H), 1.16 (d, *J* = 6.8 Hz, 3H), 1.06 (s, 3H), 1.02 (s, 3H), 0.85 (d, *J* = 7.3 Hz, 3H). ^13^C NMR (101 MHz, CDCl_3_) δ 210.06, 154.56, 149.98, 135.48, 117.34, 102.23, 90.15, 86.85, 70.93, 70.75, 63.98, 60.43, 57.77, 48.32, 46.89, 45.43, 40.66, 38.66, 35.32, 33.07, 33.04, 27.01, 26.10, 26.01, 23.22, 18.48, 14.53, 8.10. HRMS (ESI) *m*/*z*: calcd for C_28_H_38_N_6_O_3_ [M + H]^+^ 507.3078, found 507.3107.

Compound **1d**. **JN1** (40 mg, 0.10 mmol) and 3-cyclohexyl-1-propyne (16 μL, 0.11 mmol) were reacted according to the general procedure. Purification by flash column chromatography (n-hexane/EtOAc 6:4) afforded **1d** (24 mg, 46%) as a white solid. R_f_ = 0.59 (n-hexane/EtOAc 1:1). ^1^H NMR (500 MHz, CDCl_3_) δ 7.52 (s, 1H), 5.52 (d, *J* = 1.0 Hz, 1H), 5.21 (d, *J* = 7.2 Hz, 1H), 4.33 (br s, 1H, OH), 3.42 (d, *J* = 11.0 Hz, 1H), 3.32 (d, *J* = 11.0 Hz, 1H), 2.84 (m, 1H), 2.62–2.52 (m, 2H), 2.15 (q, *J* = 7.3 Hz, 1H), 1.99–1.91 (m, 2H), 1.85 (m, 2H), 1.71–1.65 (m, 6H), 1.61–1.52 (m, 1H), 1.35 (s, 3H), 1.25 (d, *J* = 6.7 Hz, 3H), 1.20–1.15 (m, 2H), 1.13 (s, 3H), 1.08 (s, 3H), 0.99–0.91 (m, 2H), 0.92 (d, *J* = 7.4 Hz, 3H). ^13^C NMR (126 MHz, CDCl_3_) δ 210.05, 150.13, 147.63, 135.39, 119.27, 102.23, 90.17, 86.95, 70.98, 70.76, 63.94, 57.93, 48.35, 47.11, 45.47, 40.64, 38.71, 37.89, 33.54, 33.19, 33.12, 27.13, 26.38, 26.10 (2C), 23.24, 18.41, 14.54, 8.12. HRMS (ESI) *m*/*z*: calcd for C_29_H_40_N_6_O_3_ [M + H]^+^ 521.3235, found 521.3267.

Compound **1e**. **JN1** (40 mg, 0.10 mmol) and phenylacetylene (12 μL, 0.11 mmol) were reacted according to the general procedure. Purification by flash column chromatography (n-hexane/EtOAc 6:4) afforded **1e** (34 mg, 68%) as a white solid. R_f_ = 0.65 (n-hexane/EtOAc 6:4). ^1^H NMR (400 MHz, CDCl_3_) δ 8.02 (s, 1H), 7.76 (d, *J* = 7.7 Hz, 2H), 7.40 (t, *J* = 7.4 Hz, 2H), 7.32 (t, *J* = 7.3 Hz, 1H), 5.56 (s, 1H), 5.19 (d, *J* = 6.9 Hz, 1H), 3.50–3.44 (br s, 1H, OH, overlapped), 3.45 (d, *J* = 11.1 Hz, 1H), 3.29 (d, *J* = 11.0 Hz, 1H), 2.89 (m, 1H), 2.12 (q, *J* = 7.2 Hz, 1H), 1.98–1.93 (m, 2H), 1.82–1.75 (m, 2H), 1.36 (s, 3H), 1.23 (d, *J* = 6.8 Hz, 3H), 1.11 (s, 3H), 1.10 (s, 3H), 0.89 (d, *J* = 7.3 Hz, 3H). ^13^C NMR (101 MHz, CDCl_3_) δ 209.69, 149.76, 148.59, 135.80, 130.28, 128.85 (2C), 128.33, 125.72 (2C), 117.89, 102.14, 90.17, 86.90, 70.91, 70.78, 64.17, 57.93, 48.39, 46.95, 45.43, 40.67, 38.60, 27.01, 23.19, 18.52, 14.53, 8.06. HRMS (ESI) *m*/*z*: calcd for C_28_H_32_N_6_O_3_ [M + H]^+^ 501.2609, found 501.2625.

Compound **1f**. **JN1** (40 mg, 0.10 mmol) and 3-phenyl-1-propyne (14 μL, 0.11 mmol) were reacted according to the general procedure. Purification by flash column chromatography (n-hexane/EtOAc 6:4) afforded **1f** (33 mg, 64%) as a white solid. R_f_ = 0.47 (n-hexane/EtOAc 4:6). ^1^H NMR (400 MHz, CDCl_3_) δ 7.40 (s, 1H), 7.29 (t, *J* = 7.7 Hz, 2H), 7.23–7.20 (m, 3H), 5.49 (s, 1H), 5.18 (d, *J* = 7.4 Hz, 1H), 4.34 (br s, 1H, OH), 4.05 (q, 2H), 3.28 (q, *J* = 11.0 Hz, 2H), 2.78 (m, 1H), 2.09 (q, *J* = 7.2 Hz, 1H), 1.95–1.85 (m, 2H), 1.85–1.73 (m, 2H), 1.31 (s, 3H), 1.20 (d, *J* = 6.7 Hz, 3H), 1.09 (s, 3H), 0.88 (s, 3H). 0.87 (d, *J* = 7.3 Hz, 3H). ^13^C NMR (101 MHz, CDCl_3_) δ 209.99, 149.94, 148.42, 138.50, 135.57, 128.75 (2C), 128.66 (2C), 126.64, 119.36, 102.09, 90.16, 86.85, 70.96, 70.75, 64.12, 57.84, 48.30, 46.91, 45.42, 40.56, 38.66, 32.33, 27.12, 23.27, 18.29, 14.58, 8.10. HRMS (ESI) *m*/*z*: calcd for C_29_H_34_N_6_O_3_ [M + H]^+^ 515.2765, found 515.2758.

Compound **2a**. **JN1** (40 mg, 0.10 mmol) and cyclopentylacetylene (26 μL, 0.22 mmol) were reacted according to the general procedure. Purification by flash column chromatography (n-hexane/EtOAc 1:1) afforded **2a** (30 mg, 51%) as a white solid. R_f_ = 0.22 (n-hexane/EtOAc 4:6). ^1^H NMR (400 MHz, CDCl_3_) δ 7.60 (s, 1H), 7.11 (s, 1H), 5.52 (s, 1H), 5.15 (d, *J* = 7.1 Hz, 1H), 5.03 (br s, 1H, OH), 4.65 (d, *J* = 12.1 Hz, 1H), 4.29 (d, *J* = 12.1 Hz, 1H), 3.13 (m, 2H), 2.83 (m, 1H), 2.22 (q, *J* = 7.4 Hz, 1H), 2.15–2.02 (m, 4H), 1.98–1.88 (m, 2H), 1.85–1.75 (m, 2H), 1.74–1.55 (m, 12H), 1.25 (s, 3H), 1.23 (d, *J* = 6.5 Hz, 3H), 1.12 (s, 3H), 0.93 (d, *J* = 7.3 Hz, 3H), 0.90 (s, 3H). ^13^C NMR (101 MHz, CDCl_3_) δ 208.40, 153.66, 151.76, 149.93, 135.76, 119.38, 117.74, 102.46, 90.25, 86.47, 71.01, 69.08, 63.99, 60.41, 55.03, 48.40, 47.06, 46.18, 41.12, 38.76, 36.84, 36.70, 33.35, 33.30, 33.17, 27.18, 25.23, 25.19, 25.12, 23.90, 21.03, 18.45, 14.47, 8.05. HRMS (ESI) *m*/*z*: calcd for C_34_H_46_N_6_O_3_ [M + H]^+^ 587.3704, found 587.3686.

Compound **2b**. **JN1** (40 mg, 0.10 mmol) and 3-cyclopentyl-1-propyne (28 μL, 0.22 mmol) were reacted according to the general procedure. Purification by flash column chromatography (n-hexane/EtOAc 1:1) afforded **2b** (32 mg, 52%) as a white solid. R_f_ = 0.31 (n-hexane/EtOAc 4:6). ^1^H NMR (400 MHz, CDCl_3_) δ 7.61 (s, 1H), 7.15 (s, 1H), 5.55 (s, 1H), 5.15 (d, *J* = 7.1 Hz, 1H), 4.69 (d, *J* = 12.1 Hz, 1H), 4.31 (d, *J* = 12.0 Hz, 2H), 4.30–4.24 (br s, 1H, OH, overlapped), 2.85 (m, 1H), 2.71 (br d, *J* = 7.4 Hz, 2H), 2.67 (br d, *J* = 7.6 Hz, 2H), 2.22–2.11 (m, 3H), 2.01–1.94 (m, 2H), 1.92– 1.80 (m, 2H), 1.79–1.67 (m, 4H), 1.64–1.55 (m, 4H), 1.54–1.43 (m, 4H), 1.27 (s, 3H), 1.25 (d, *J* = 6.6 Hz, 3H), 1.23–1.16 (m, 4H), 1.14 (s, 3H), 0.95 (d, *J* = 7.2 Hz, 3H), 0.88 (s, 3H). ^13^C NMR (101 MHz, CDCl_3_) δ 208.08, 149.92, 148.67, 147.03, 135.74, 120.72, 119.13, 102.29, 90.23, 86.42, 70.91, 69.04, 63.93, 54.96, 48.49, 46.91, 46.19, 41.09, 39.97, 39.74, 38.74, 32.62, 32.60, 32.43 (2C), 31.87, 31.67, 27.25, 25.10 (2C), 25.05, 25.03, 23.72, 18.50, 14.46, 8.05. HRMS (ESI) *m*/*z*: calcd for C_36_H_50_N_6_O_3_ [M + H]^+^ 615.4017, found 615.4042.

Compound **2c**. **JN1** (40 mg, 0.10 mmol) and cyclohexylacetylene (28 μL, 0.22 mmol) were reacted according to the general procedure. Purification by flash column chromatography (n-hexane/EtOAc 1:1) afforded **2c** (34 mg, 55%) as a white solid. R_f_ = 0.28 (n-hexane/EtOAc 4:6). ^1^H NMR (400 MHz, CDCl_3_) δ 7.59 (s, 1H), 7.08 (s, 1H), 5.51 (s, 1H), 5.12 (d, *J* = 7.0 Hz, 1H), 4.97 (br s, 1H, OH), 4.61 (d, *J* = 12.2 Hz, 1H), 4.32 (d, *J* = 12.2 Hz, 1H), 2.91–2.78 (m, 1H), 2.78–2.62 (m, 2H), 2.22 (m, 1H), 2.09–1.95 (m, 4H), 1.93–1.79 (m, 2H), 1.69–1.62 (m, 8H), 1.35–1.31 (m, 8H), 1.26 (s, 3H), 1.25–1.22 (m, 2H), 1.22 (d, *J* = 6.9 Hz, 3H), 1.13 (s, 3H), 0.92 (d, *J* = 7.3 Hz, 3H), 0.88 (s, 3H). ^13^C NMR (101 MHz, CDCl_3_) δ 208.33, 154.61, 152.76, 149.98, 135.70, 118.82, 117.53, 102.44, 90.21, 86.49, 70.98, 69.04, 63.96, 60.41, 54.96, 48.37, 46.95, 46.14, 41.16, 38.79, 35.35, 35.18, 33.23, 33.13, 32.97, 32.85, 27.17, 26.11 (2C), 26.01, 25.99, 23.88, 18.49, 14.45, 14.17, 8.04. HRMS (ESI) *m*/*z*: calcd for C_36_H_50_N_6_O_3_ [M + H]^+^ 615.4017, found 615.4041.

Compound **2d**. **JN1** (40 mg, 0.10 mmol) and 3-cyclohexyl-1-propyne (32 μL, 0.22 mmol) were reacted according to the general procedure. Purification by flash column chromatography (n-hexane/EtOAc 1:1) afforded **2d** (36 mg, 57%) as a white solid. R_f_ = 0.30 (n-hexane/EtOAc 1:1). ^1^H NMR (400 MHz, CDCl_3_) δ 7.60 (s, 1H), 7.13 (s, 1H), 5.54 (s, 1H), 5.12 (d, *J* = 6.9 Hz, 1H), 4.83 (br s, 1H, OH), 4.67 (d, *J* = 12.1 Hz, 1H), 4.30 (d, *J* = 12.1 Hz, 1H), 2.84 (m, 1H), 2.67–2.44 (m, 4H), 2.25–2.15 (m, 1H), 2.09 –2.01 (m, 2H), 1.98–1.92 (m, 1H), 1.88–1.81 (m, 1H), 1.74–1.54 (m, 14H), 1.26 (s, 3H), 1.23 (d, *J* = 6.5 Hz, 3H), 1.20–1.09 (m, 4H), 1.10 (s, 3H), 0.93 (d, *J* = 7.3 Hz, 3H), 0.94–0.85 (m, 4H), 0.87 (s, 3H). ^13^C NMR (101 MHz, CDCl_3_) δ 208.32, 149.83, 147.50, 145.91, 135.85, 121.11, 119.56, 102.38, 90.27, 86.40, 70.96, 69.03, 63.96, 54.90, 48.43, 46.85, 46.14, 41.08, 38.75, 37.91, 37.89, 33.54, 33.33, 33.13, 33.07 (2C), 33.00, 27.24, 26.45, 26.38, 26.16, 26.15, 26.10 (2C), 23.72, 18.55, 14.49, 8.05. HRMS (ESI) *m*/*z*: calcd for C_38_H_54_N_6_O_3_ [M + H]^+^ 643.4330, found 643.4310.

Compound **2e**. **JN1** (40 mg, 0.10 mmol) and phenylacetylene (24 μL, 0.22 mmol) were reacted according to the general procedure. Purification by flash column chromatography (n-hexane/EtOAc 1:1) afforded **2e** (26 mg, 44%) as a white solid. R_f_ = 0.41 (n-hexane/EtOAc 6:4). ^1^H NMR (400 MHz, CDCl_3_/CD_3_OD) δ 8.19 (s, 1H), 7.88 (br d, *J* = 8.3 Hz, 2H), 7.87 (s, 1H), 7.77 (br d, *J* = 7.5 Hz, 2H), 7.43–7.39 (m, 3H), 7.40–7.29 (m, 3H), 5.63 (s, 1H), 5.15 (d, *J* = 6.8 Hz, 1H), 4.93 (d, *J* = 12.1 Hz, 1H), 4.48 (d, *J* = 12.1 Hz, 1H), 3.36 (br s, 1H, OH), 3.00 (m, 1H), 2.24–2.20 (m, 1H), 2.17–2.07 (m, 2H), 2.05–1.99 (m, 1H), 1.81 (t, *J* = 12.6 Hz, 1H), 1.35 (d, *J* = 6.7 Hz, 3H), 1.30 (s, 3H), 1.20 (s, 3H), 1.02 (s, 3H), 1.00 (d, *J* = 7.5 Hz, 3H). ^13^C NMR (101 MHz, CDCl_3_/CD_3_OD) δ 208.87, 149.78, 148.41, 147.15, 135.98, 130.15, 130.06, 128.87 (2C), 128.74 (2C), 128.35, 128.14, 125.67 (2C), 125.60 (2C), 119.91, 118.47, 102.38, 89.94, 86.53, 70.99, 69.37, 64.14, 55.10, 48.18, 46.68, 45.95, 41.16, 38.68, 26.79, 23.77, 18.31, 14.01, 7.73. HRMS (ESI) *m*/*z*: calcd for C_36_H_38_N_6_O_3_ [M + H]^+^ 603.3078, found 603.3089.

Compound **2f**. **JN1** (40 mg, 0.10 mmol) and 3-phenyl-1-propyne (28 μL 0.22 mmol) were reacted according to the general procedure. Purification by flash column chromatography (n-hexane/EtOAc 1:1) afforded **2f** (30 mg, 47%) as a white solid. R_f_ = 0.21 (n-hexane/EtOAc 4:6). ^1^H NMR (400 MHz, CDCl_3_) δ 7.41 (s, 1H), 7.21–7.10 (m, 10H), 7.03 (s, 1H), 5.41 (s, 1H), 5.05 (d, *J* = 7.3 Hz, 1H), 5.05–4.98 (br s, 1H, OH, overlapped), 4.52 (d, *J* = 12.1 Hz, 1H), 4.16 (d, *J* = 12.1 Hz, 1H), 4.07–3.88 (m, 4H), 2.80–2.64 (m, 1H), 2.09 (q, *J* = 7.2 Hz, 1H), 1.95–1.85 (m, 2H), 1.83–1.70 (m, 2H), 1.16 (s, 3H), 1.10 (d, *J* = 6.6 Hz, 3H), 0.99 (s, 3H), 0.80 (d, *J* = 7.2 Hz, 3H), 0.63 (s, 3H). ^13^C NMR (101 MHz, CDCl_3_) δ 208.44, 149.84, 148.39, 146.44, 139.08, 138.71, 135.84, 128.81 (2C), 128.70 (2C), 128.66 (2C), 128.61 (2C), 126.45, 126.43, 121.63, 119.55, 102.30, 90.26, 86.31, 70.99, 69.27, 64.10, 55.00, 48.38, 46.83, 46.09, 40.94, 38.72, 32.37, 32.09, 27.21, 24.04, 18.34, 14.54, 8.02. HRMS (ESI) *m*/*z*: calcd for C_38_H_42_N_6_O_3_ [M + H]^+^ 631.3391, found 631.3398.

Compound **2g**. **JN1** (40 mg, 0.10 mmol) and 4-phenyl-1-butyne (31 μL 0.22 mmol) were reacted according to the general procedure. Purification by flash column chromatography (n-hexane/EtOAc 1:1) afforded **2g** (38 mg, 58%) as a white solid. R_f_ = 0.47 (n-hexane/EtOAc 4:6). ^1^H NMR (400 MHz, CDCl_3_) δ 7.64 (s, 1H), 7.30–7.06 (m, 10H), 7.01 (s, 1H), 5.54 (s, 1H), 5.17 (d, *J* = 7.0 Hz, 1H), 4.95 (br s, 1H, OH), 4.78 (d, *J* = 12.1 Hz, 1H), 4.24 (d, *J* = 12.1 Hz, 1H), 3.09–2.91 (m, 8H), 2.87–2.82 (m, 1H), 2.22 (q, *J* = 7.1 Hz, 1H), 2.09–1.98 (m, 2H), 1.96–1.83 (m, 2H), 1.29 (s, 3H), 1.26 (d, *J* = 5.9 Hz, 3H), 1.05 (s, 3H), 0.93 (d, *J* = 7.2 Hz, 3H), 0.87 (s, 3H). ^13^C NMR (101 MHz, CDCl_3_) δ 208.26, 149.86, 148.26, 146.34, 141.30, 141.21, 135.83, 128.56 (2C), 128.45 (2C), 128.38 (2C), 128.33 (2C), 126.08, 125.98, 121.22, 119.38, 102.39, 90.32, 86.47, 71.01, 69.11, 64.04, 54.96, 48.43, 46.97, 46.17, 41.10, 38.80, 35.52, 35.49, 27.78, 27.55, 27.09, 23.83, 18.48, 14.51, 8.03. HRMS (ESI) *m*/*z*: calcd for C_40_H_46_N_6_O_3_ [M + H]^+^ 659.3704, found 659.3682.

### 3.4. Biology

#### 3.4.1. Parasite and Cell Culture

The *T. cruzi* Cl-B5 transfected strain, kindly provided by the Department of Parasitology, Complutense University of Madrid (Madrid, Spain), was used for epimastigote assays, whereas the *T. cruzi* Tulahuen transfected strain, kindly provided by the Instituto de Investigaciones en Ingeniería Genética y Biología Molecular (INGEBI, Buenos Aires, Argentina), was employed for amastigote assays. Parasites stably transfected with the *Escherichia coli* β-galactosidase gene (lacZ) were cultured at 28 °C in liver infusion tryptose (LIT) medium supplemented with 10% fetal bovine serum (FBS), penicillin, and streptomycin, as previously described [45], and harvested during the exponential growth phase. Metacyclic trypomastigotes were obtained by differentiation of epimastigotes in modified Grace medium [46]. L929 and Vero cell lines, kindly provided by the Department of Parasitology, Complutense University of Madrid (Madrid, Spain), were maintained in Minimal Essential Medium (Sigma-Aldrich, St. Louis, MO, USA), and Dulbecco’s Modified Eagle Medium (Sigma-Aldrich, St. Louis, MO, USA), respectively. Both media were supplemented with 5% heat-inactivated FBS, penicillin G (100 U/mL), and streptomycin (100 µg/mL). For the assays, pre-confluent cells were detached with trypsin and used as required. All cell cultures were maintained at 37 °C in a humidified atmosphere containing 5% CO_2_.

#### 3.4.2. Epimastigote Susceptibility Assay

The screening assay was performed in 96-well microplates (Nunc, Roskilde, Denmark) using exponentially growing cultures, as previously described [47]. Briefly, *T. cruzi* Cl-B5 β-gal epimastigotes were seeded at 2.5 × 10^5^ parasites/mL in a final volume of 200 µL per well. Plates were incubated with test compounds at 28 °C for 72 h. Subsequently, 50 µL of chlorophenol red β-D-galactopyranoside (CPRG, Sigma-Aldrich, St. Louis, MO, USA) solution was added to reach a final concentration of 200 µM. Plates were further incubated at 37 °C for 4 h, and absorbance was measured at 595 nm [47]. Benznidazole (Sigma-Aldrich, St. Louis, MO, USA) was used as the reference drug. Each concentration was tested in triplicate, and two independent experiments were performed. Compound efficacy was expressed as the IC_50_ value, calculated by nonlinear regression analysis. IC_50_ values are reported as mean ± SD.

#### 3.4.3. Amastigote Susceptibility Assay

Anti-amastigote activity was evaluated using a modified colorimetric assay [48] with Vero cells as host cells and metacyclic trypomastigotes of *T. cruzi* Tulahuen β-gal as infective parasites. Vero fibroblasts were seeded in 48-well plates at 5 × 10^4^ cells per well and allowed to adhere. Metacyclic trypomastigotes were added at a parasite-to-cell ratio of 2:1 and incubated for 24 h at 37 °C in a humidified atmosphere containing 5% CO_2_. After infection, cells were washed twice with PBS to remove non-infective trypomastigotes. Test compounds were added in triplicate (final volume 450 µL per well), and plates were incubated for 72 h under the same conditions. Subsequently, 100 µL of CPRG solution (100 µM final concentration) in 0.3% Triton X-100 was added to each well. After 4 h at 37 °C, β-galactosidase activity was determined by measuring the optical density at 595 nm using a microplate reader (BioTek Instruments, Winooski, VT, USA). Anti-amastigote activity was expressed as percentage inhibition relative to untreated infected controls according to the following equation: %AA = 100 − (OD_exp/OD_ctrl) × 100. Background absorbance (NCTC-929 cells only) was subtracted from all values. IC_50_ values are reported as mean ± SD.

#### 3.4.4. Cytotoxicity Assay

Cell viability was assessed using a resazurin-based colorimetric assay with minor modifications as previously described [49]. L929 cells were seeded in 96-well plates (Nunc) at a density of 1 × 10^4^ cells per well in 100 µL of growth medium and incubated overnight at 37 °C in a humidified atmosphere containing 5% CO_2_. The medium was then removed and replaced with 200 µL of fresh medium containing the test compounds, followed by incubation for 48 h. Subsequently, 20 µL of resazurin solution (2 mM) was added to each well, and plates were incubated for an additional 4 h. Resazurin reduction was quantified by measuring absorbance at 490 and 595 nm. Background absorbance was subtracted from all values. Each concentration was tested in triplicate, and medium and drug controls were included in each experiment. Cytotoxicity was expressed as IC_50_ values, calculated by nonlinear regression analysis and reported as mean ± SD from two independent experiments.

## 4. Conclusions

Jatrophone was successfully used as a versatile scaffold to generate mono- and bis-triazole derivatives via CuAAC, enabling systematic exploration of structure–activity and selectivity relationships. Triazole functionalization modulated cytotoxicity and antiparasitic activity, with methylene-linked substituents improving selectivity in extracellular assays. Nevertheless, most compounds showed limited selectivity in intracellular amastigote models, highlighting the challenges of translating extracellular activity into physiologically relevant contexts. Overall, these results demonstrate that triazole derivatization is a promising strategy to tune the biological profile of jatrophone and supports its continued exploration as a lead-like scaffold for antitrypanosomal drug discovery.

## Figures and Tables

**Table 1:** In vitro activity against *Trypanosoma cruzi* epimastigotes, cytotoxicity toward L929 fibroblasts, and selectivity index (SI). IC_50_ values are expressed in µM.

Compound		*T. cruzi* (CL β-gal) IC_50_ (µM)	L929 Cells IC_50_ (µM)	Selectivity Index (SI)
**JAT**		1.7 ± 0.28	5.6 ± 0.23	3.3
**JN1** 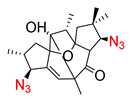		4.1 ± 0.21	116.9 ± 0.85	28.5
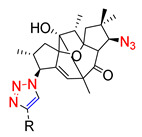				
**1a**	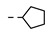	16.3 ± 0.54	35.4 ± 0.54	2.2
**1b**	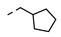	4.8 ± 0.21	>256 *	≥53
**1c**	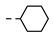	22.1 ± 0.28	35.6 ± 0.62	1.6
**1d**	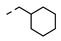	127.0 ± 0.85	>256 *	≥2.0
**1e**	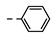	15.5 ± 0.42	32.5 ± 0.94	2.1
**1f**	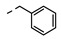	22.9 ± 0.35	>256 *	≥11.2
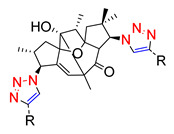				
**2a**	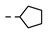	36.1 ± 1.24	93.8 ± 1.10	2.6
**2b**	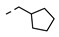	20.3 ± 0.57	36.0 ± 0.52	1.8
**2c**	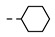	31.3 ± 0.35	56.2 ± 0.74	1.8
**2d**	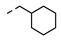	24.2 ± 0.66	>256 *	≥10.6
**2e**	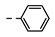	23.1 ± 0.32	>256 *	≥11.1
**2f**	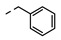	61.7 ± 0.70	>256 *	≥4.1
**2g**	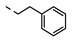	26.6 ± 0.68	>256 *	≥9.6
Benznidazole		54.7 ± 1.30	769.0 ± 3.45	14.1

* No cytotoxicity observed at the highest concentration tested (256 µM). Values are expressed as mean ± SD from two independent experiments performed in triplicate.

**Table 2:** In vitro antitrypanosomal activity against *T. cruzi* Tulahuen β-galactosidase-expressing intracellular amastigotes in Vero cells, cytotoxicity toward Vero cells, and selectivity index (SI). IC_50_ values are expressed in µM.

Compound	*T. cruzi* Tulahuen β-gal/Vero Amastigotes IC_50_ (µM)	Vero Cells IC_50_ (µM)	Selectivity Index (SI)
**JN1**	1.6 ± 0.23	0.7 ± 0.58	0.5
**1b**	9.2 ± 0.54	8.0 ± 0.49	0.9
**1f**	10.8 ± 0.41	5.0 ± 0.67	0.5
**2d**	3.5 ± 0.43	5.8 ± 0.51	1.6
**2e**	4.6 ± 0.24	7.1 ± 0.46	1.5
**2g**	10.4 ± 0.74	3.9 ± 0.62	0.4
Benznidazole	3.2 ± 0.49	>256 *	>80

* No cytotoxicity observed at the highest concentration tested (256 µM). Values are expressed as mean ± SD from two independent experiments performed in triplicate.

## Data Availability

The original contributions presented in this study are included in the article/Supplementary Material. Further inquiries can be directed to the corresponding author.

## Supplementary Materials

The following supporting information can be downloaded at https://www.mdpi.com/article/10.3390/ph19050801/s1: Figures S1–S51: NMR and HREIMS spectra of synthesized compounds. Scheme S1: Proposed mechanism for the formation of **JN1** from jatrophone. Table S1: ^1^H and ^13^C NMR spectroscopic data for **JN1** recorded in CDCl_3_. Structural Assignment of Mono-Triazole **1d**. Table S2: Key COSY correlations supporting the structure of **1d**. Regioselectivity and steric interpretation. NOESY analysis and relative stereochemistry. Table S3: ^1^H and ^13^C NMR spectroscopic data for **JN1** and **1d** in CDCl_3_. Table S4: Physicochemical and pharmacokinetic descriptors calculated with SwissADME. Table S5: Systematic IUPAC names and SMILES of synthesized compounds.
