# Supplementary material for: Triazole-Functionalized Jatrophone Derivatives as Antiprotozoal Agents Against Trypanosoma cruzi: Synthesis, Biological Evaluation and Structure—Activity Relationships
Source: Pharmaceuticals (Basel). 2026 May 21;19(5):801. doi: 10.3390/ph19050801 (PMC13210208; doi:10.3390/ph19050801)
Supplement: Supplementary file 1 [file pharmaceuticals-19-00801-s001.zip › pharmaceuticals-4297843-supplementary.pdf]

Supporting Information

Triazole-functionalized jatrophone derivatives as antiprotozoal agents against *Trypanosoma cruzi*: synthesis, biological evaluation and structure–activity relationships

MarianoWalter Pertino [a],* Patricio Carreño Gonzalez [a], Camila Venegas [b], Guillermo Schmeda-Hirschmann [a], Celeste Vega Gómez [c], Miriam Rolón [c], Antonieta Rojas de Arias [c].

[a] Instituto de Química de Recursos Naturales, Universidad de Talca, Campus Lircay, Talca 3480094, Chile.

[b] Escuela de ingeniería en biotecnología, Facultad de ciencias agrarias y forestales, Universidad católica del Maule, Talca, Chile.

[c] Centro para el Desarrollo de la Investigación Científica (CEDIC), Manduvirá 635, Asunción CP 1255, Paraguay.

* Correspondence: mwalter@utalca.cl; Tel.: +56-71-2418866

Table of Contents

Proposed Mechanism for the Formation of **JN1** 2

Structural Characterization of **JN1** 2

Table S1. ^{1}H and ^{13}C NMR spectroscopic data for **JN1** 3

Structural Assignment of Mono-Triazole **1d** 4

Table S2. Key COSY Correlations Supporting the Structure of **1d** 4

Regioselectivity and Steric Interpretation 4

NOESY Analysis and Relative Stereochemistry 5

Table S3. ^{1}H and ^{13}C NMR spectroscopic data for **JN1** and **1d** 6

Figures S1–S51. NMR and HREIMS spectra of synthesized compounds 7–45

Table S4. Physicochemical and pharmacokinetic descriptors calculated with SwissADME........46

Table S5. Systematic IUPAC names and SMILES of synthesized compounds …........ 47

Proposed Mechanism for the Formation of JN1

The proposed mechanism for the formation of **JN1** is shown in Scheme S1 and is based on the conjugated cascade cyclization mechanism previously reported by Hady et al. for nucleophilic additions to jatrophone derivatives. In the present case, azide is proposed to act as the nucleophile, promoting sequential Michael-type additions to the electrophilic α,β-unsaturated system of jatrophone under mildly acidic conditions. The initial nucleophilic attack likely induces electron redistribution within the conjugated framework, facilitating a second azide addition followed by protonation to afford the diazido derivative **JN1**. This proposal should be considered a plausible mechanistic rationale based on literature precedent and the observed reaction outcome rather than definitive mechanistic proof.

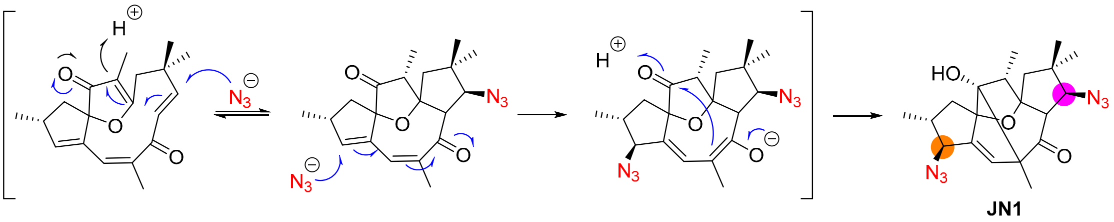

Scheme S1. Proposed mechanism for the formation of **JN1** from jatrophone (adapted from Hady et al. for thiol addition).

Structural Characterization of JN1

The structural characterization of the diazido precursor **JN1** was established primarily through HSQC and HMBC experiments, which enabled assignment of the protonated carbons and confirmation of the modified jatrophone framework. The HMQC spectrum allowed for direct correlation of proton and carbon resonances, particularly for the heteroatom-substituted methines H-3, H-8, and H-9, which correlated with carbons in the δC 57–71 ppm region. The olefinic proton H-5 correlated with the sp2 carbon at δC 135.31, supporting preservation of the α,β-unsaturated system of the jatrophone scaffold.

The HMBC spectrum provided key long-range correlations supporting the proposed connectivity. Correlations from H-5 to C-17, C-3, C-6, C-14, C-15, and C-4 confirmed the α,β-unsaturated framework, while correlations involving methyl groups H-17 to C-6 and C-14, H-19 to C-18, C-10, C-11 and C-9, and H-20 to C-13, C12 and C-14 further supported the preservation of the diterpenoid skeleton after azidation (Figure S5).

Table S1. ^{1}H and ^{13}C NMR spectroscopic data for **JN1** recorded in $CDCl_3$ (δ in ppm, *J* in Hz).

	^{1}H	^{13}C
1 α	1.91 – 1.86 m*	38.25 t
1 β	1.57 dd (13.6; 9.7)*	
2	2.64 m	43.15 d
3	3.70 d (5.5)	64.57 d
4	-	150.96 s
5	5.60 s	135.31 d
6	-	70.48 s
7	-	210.67 s
8	3.68 d (10.9)	57.29 d
9	3.25 d (11,0)	70.94 d
10	-	40.57 s
11 α	1.91 – 1.86 m*	45.47 t
11 β	1.74 d (15.0)*	
12	-	86.35 s
13	2.00 q (7.3)	48.69 d
14	-	90.28 s
15	-	101.22 s
16	1.09 d (5.3)	18.68 q
17	1.34 s	14.59 q
18	1,08 s	27.23 q
19	1,01 s	22.88 q
20	0.86 d (7.3)	8.04 q
OH	not observed	-

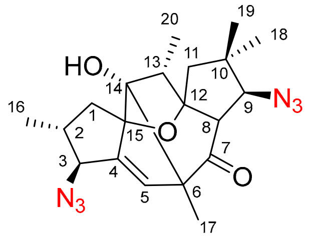

*The stereochemical assignment of H-1α/H-1β and H-11α/H-11β should be considered tentative, as these signals may be interchangeable. These proton signals were assigned based on HSQC correlations.

Structural Assignment of Mono-Triazole 1d

Formation of mono-triazole derivative **1d** was confirmed by the appearance of the characteristic triazole proton at δH 7.52 together with the corresponding triazole carbons at δC 119.19 and 147.63. Comparison with the diazido precursor **JN1** showed that only one azide-bearing methine environment underwent a significant downfield shift after cycloaddition, whereas the second azide-bearing position remained within the heteroatom-substituted region. These observations support selective mono-functionalization.

The COSY spectrum provided additional connectivity information supporting the assigned structure. Correlations of H-2 with H-16, H-1, and H-3 confirmed preservation of the C1–C2–C3 spin system. Correlations between H-9 and H-8 supported the vicinal relationship between the two heteroatom-substituted methines, while correlations between H-13 and H-20 confirmed preservation of the aliphatic region of the jatrophone skeleton. Importantly, COSY correlations between H-3′ and H-4′ confirmed incorporation of the cyclohexylmethyl fragment introduced during the CuAAC reaction. (Figure S18)

Table S2. Key COSY Correlations Supporting the Structure of **1d**

Observed Correlation	Assignment	Structural Interpretation
H-2 ↔ H-16 / H-1 / H-3	C1–C2–C3 fragment	Confirms preservation of diterpenoid connectivity
H-9 ↔ H-8	Vicinal heteroatom-substituted methines	Supports relative arrangement of azide/oxygenated centers
H-13 ↔ H-20	Aliphatic region	Confirms integrity of jatrophone skeleton
H-3′ ↔ H-4′	Cyclohexylmethyl fragment	Confirms incorporation of CuAAC substituent

Regioselectivity and Steric Interpretation

The preferential formation of a single mono-triazole regioisomer can be rationalized mainly by steric and conformational factors associated with the rigid jatrophone scaffold. Visual inspection of a minimized three-dimensional model suggests that one azide group is located close to a sterically congested region containing a gem-dimethyl-substituted carbon center, which likely hinders the approach of the copper–acetylide species during the CuAAC reaction.

In contrast, the second azide group is more sterically accessible and therefore reacts preferentially during the first cycloaddition step, leading to selective formation of the mono-triazole derivatives under mild conditions.

Preliminary Gasteiger charge analysis showed very similar charge distributions for both azide groups, suggesting that electronic effects are not the principal factor controlling selectivity. Thus, the observed regioselectivity is better explained by steric accessibility and conformational constraints rather than intrinsic electronic differentiation between the two azides.
These observations should be interpreted as a qualitative steric rationale rather than a definitive mechanistic proof.

NOESY Analysis and Relative Stereochemistry

The relative stereochemistry of the compound was further supported by NOESY analysis combined with a minimized three-dimensional model generated in Chem3D using the MM2 force field (total energy = 147.1573 kcal/mol).

H-3 (δ 3.30 ppm) showed strong NOESY correlations with H-16 (δ 1.25 ppm) and H-1 (δ 1.90–1.80 ppm), with estimated interproton distances of approximately 2.5 and 3.0 Å, respectively (Figure S23). These correlations are consistent with the spatial proximity of these protons and support their orientation toward the same molecular face. A weaker NOESY correlation was also observed between H-3 and H-2 (δ 2.84 ppm), despite H-2 being located on the opposite face of the five-membered ring. This weak interaction may arise from conformational flexibility and the close spatial arrangement imposed by the rigid fused-ring system, particularly considering that H-16, attached to the same carbon as H-2, is oriented toward the same face as H-3.

Additional diagnostic NOESY correlations were observed for the second stereochemical region. H-9 correlated with H-20 and H-18, whereas H-8 showed a correlation with H-19. The calculated interproton distances (approximately 2.3–2.4 Å) are consistent with experimentally observable NOE interactions and support the proposed relative configuration of the jatrophone-derived scaffold (Figure S21).

Table S3. ^{1}H and ^{13}C NMR spectroscopic data for **JN1** and **1d** in $CDCl_3$ (δ in ppm, *J* in Hz).

	^{1}H		^{13}C	
	**JN1**	**1d**	**JN1**	**1d**
1 α	1.91 – 1.86 m*	1.99-1.91 m*	38.25 t	38.71 t
1 β	1.57 dd (13.6; 9.7) *	1.57 m*		
2	2.64 m	2.84 m	43.15 d	47.11 t*
3	3.70 d (5.5)	5.21 d (7.2)	64.57 d	63.94 d
4	-	-	150.96 s	150.13 s
5	5.60 s	5.52 d (1.0)	135.31 d	135.39 d
6	-	-	70.48 s	70.76 s
7	-	-	210.67 s	210.05 s
8	3.68 d (10.9)	3.42 (11.0)	57.29 d	57.93 d
9	3.25 d (11,0)	3.32 (11.0)	70.94 d	70.98 d
10	-	-	40.57 s	40.64 s
11 α	1.91 – 1.86 m*	1.99-1.91 m*	45.47 t	45.47 t*
11 β	1.74 d (15.0) *	1.71-1.65 m *		
12	-	-	86.35 s	86.95 s
13	2.00 q (7.3)	2.15 q (7.3)	48.69 d	48.35 d
14	-	-	90.28 s	90.17 s
15	-	-	101.22 s	102.23 s
16	1.09 d (5.3)	1.25 d (6.7)	18.68 q	18.41 q
17	1.34 s	1.35 s	14.59 q	14.54 q
18	1,08 s	1.13 s	27.23 q	27.13 q
19	1,01 s	1.08 s	22.88 q	23.24 q
20	0.86 d (7.3)	0.92 d (7.4)	8.04 q	8.12 q
OH	Not observed	4.33 br s	-	-
1'	-	7.52 s	-	119.19 d
2'	-	-	-	147.63 s
3'	-	2,62-2,52 m	-	37.89 t
4'	-	1.61-1.52 m	-	47.11 d

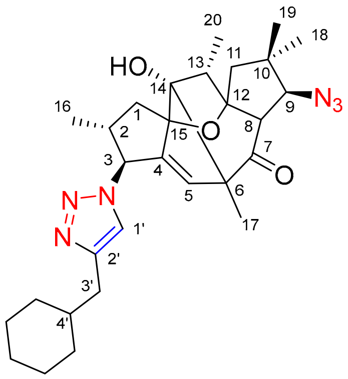

*The assignments of carbons C-2 and C-11 in compound **1d** are tentative and may be interchangeable.

The proton and carbon signals corresponding to the cyclohexane ring and C-13 were not individually assigned, as additional two-dimensional NMR experiments would be required to ensure an unambiguous assignment. Nevertheless, the number and overall pattern of the observed ^{1}H and ^{13}C signals are fully consistent with the proposed structure of compound **1d**.

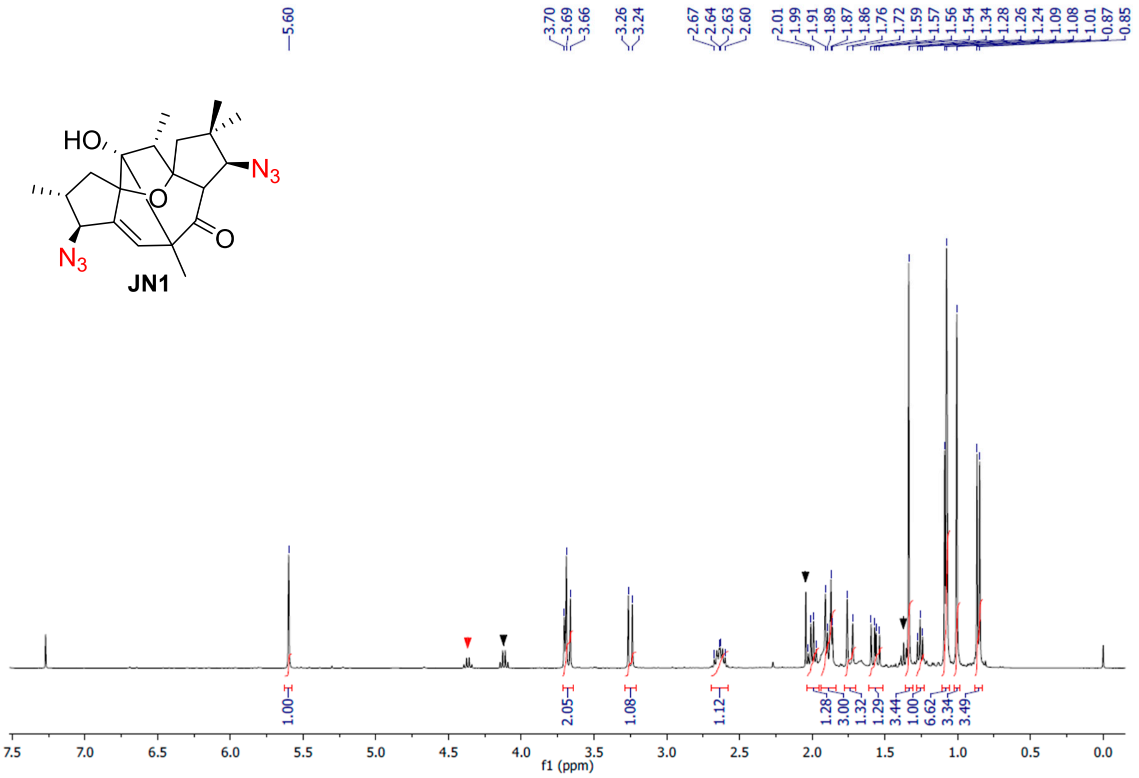

Figure S1. ^{1}H NMR spectra in $CDCl_3$ of compound **JN1**.

Note**:** Residual signals of ethyl acetate (EtOAc), used during purification, are observed in the spectra (δ ≈ 4.12, 2.05, and 1.25 ppm; black arrows). A minor signal at δ 4.4 ppm (red arrow) corresponds to an unidentified impurity with very low integration (~0.21 H), indicating trace presence without impact on structural characterization.

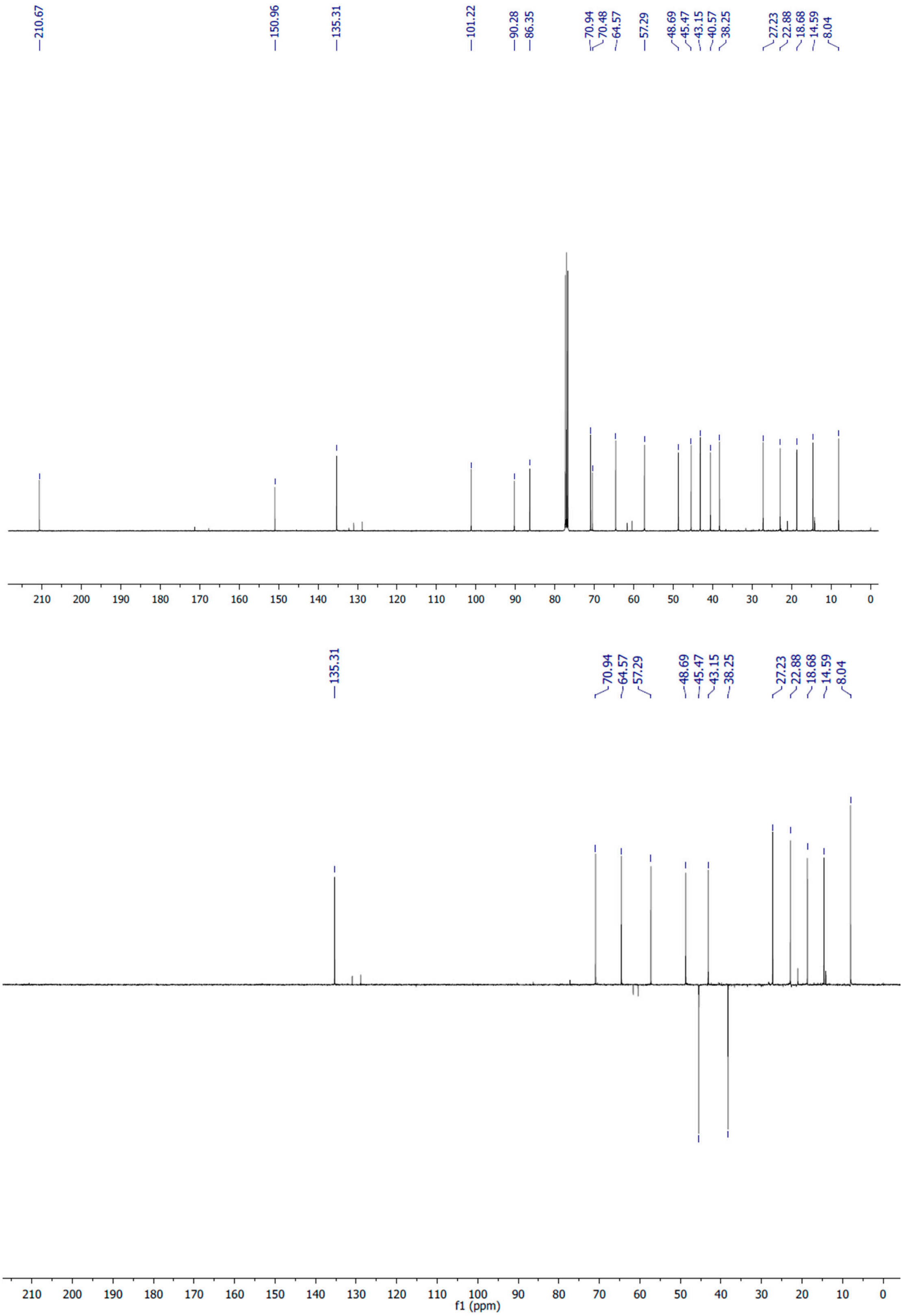

Figure S2. ^{13}C NMR and DEPT-135 spectra in $CDCl_3$ of compound **JN1**.

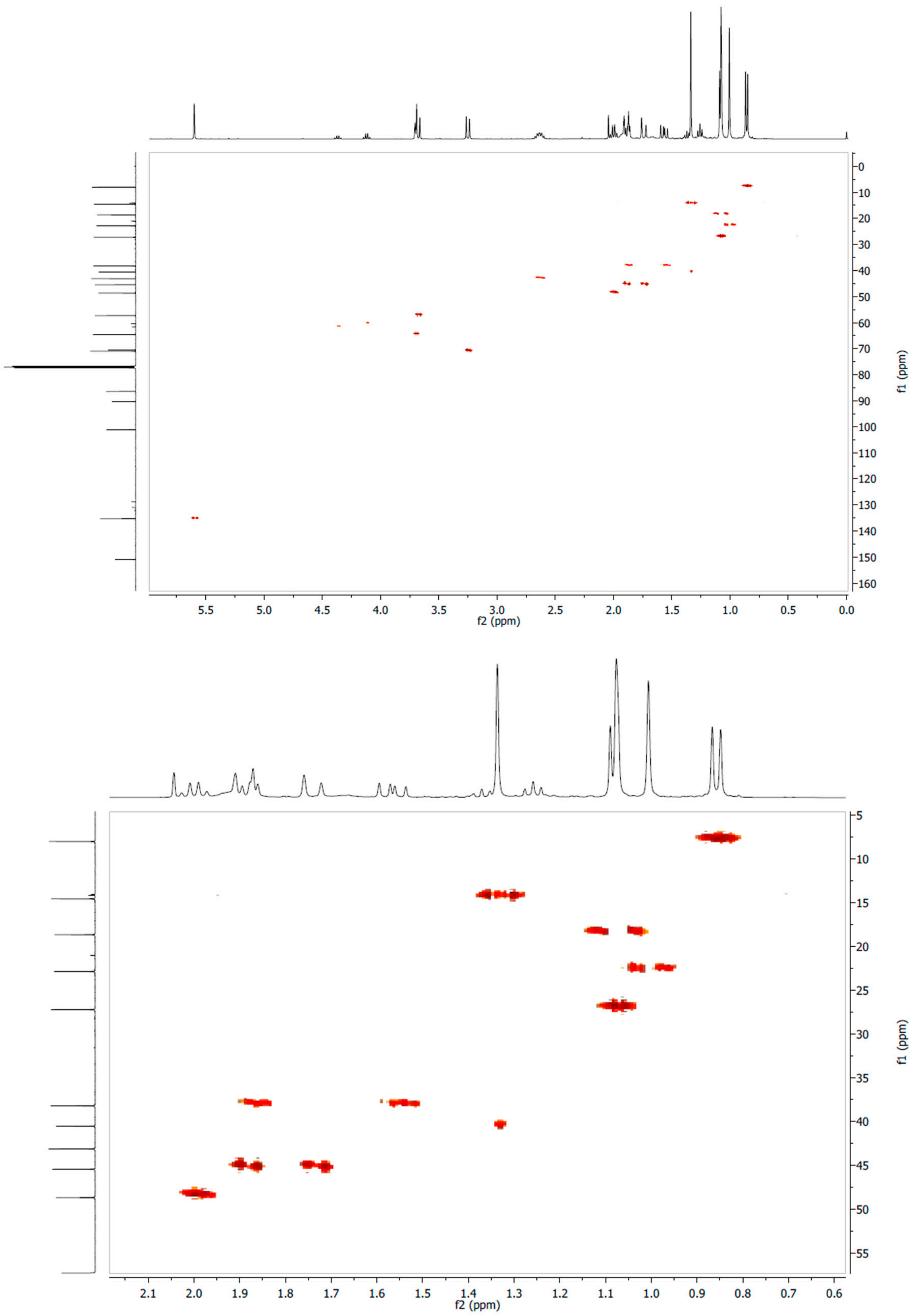

Figure S3. HSQC spectra in $CDCl_3$ of compound **JN1**.

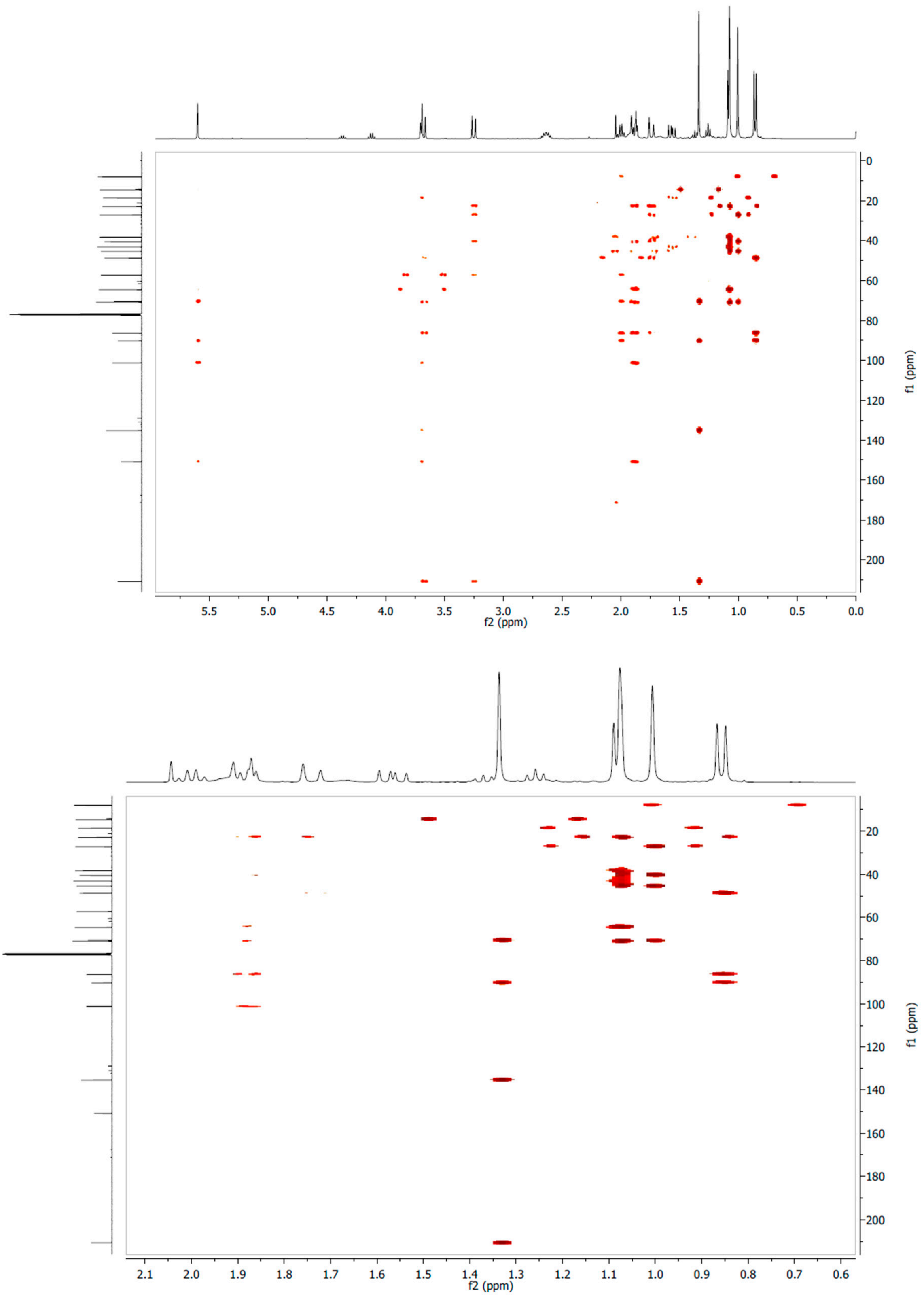

Figure S4. HMBC spectra in $CDCl_3$ of compound **JN1**.

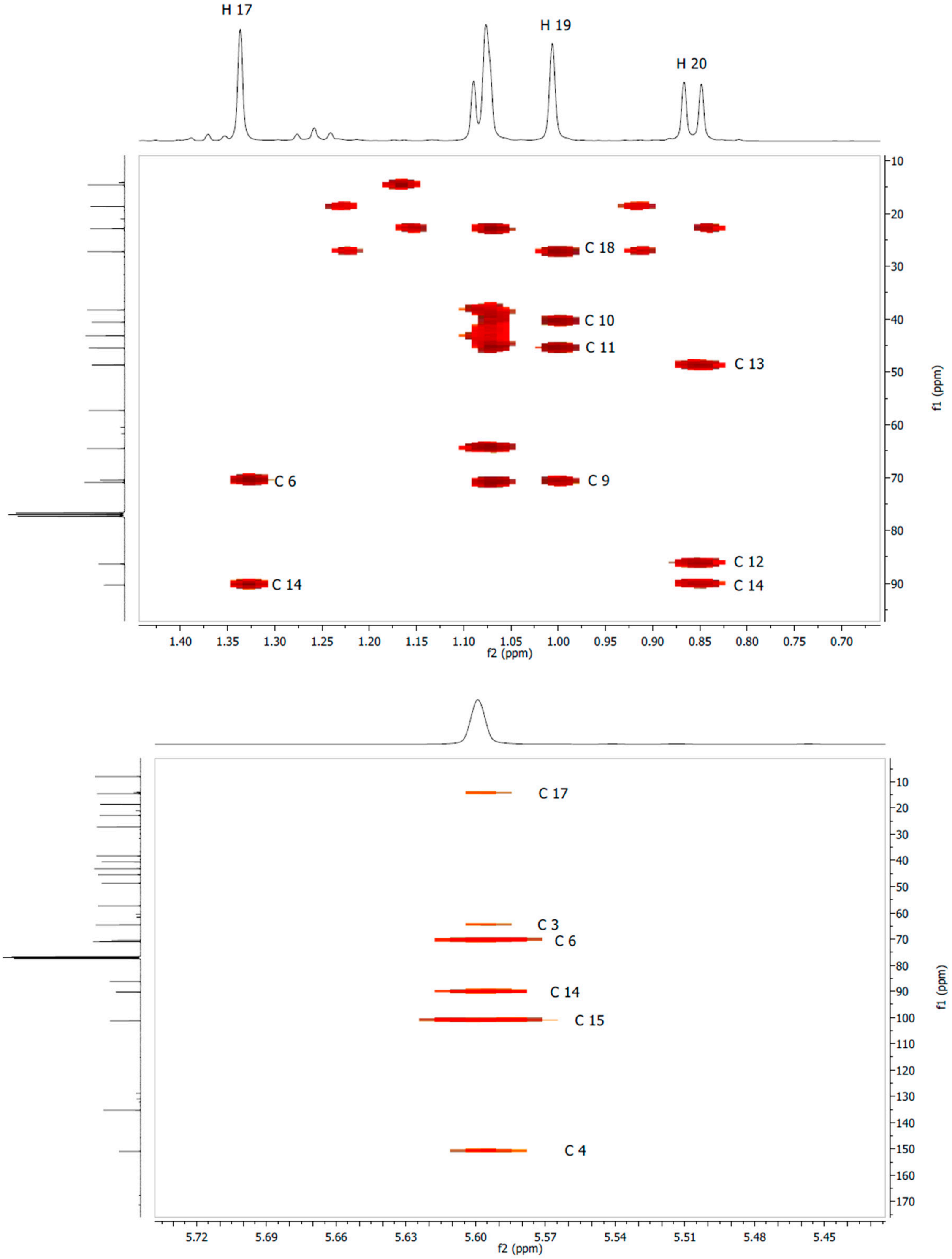

Figure S5. Key HMBC correlations observed in the $CDCl_3$ NMR spectra of compound **JN1**

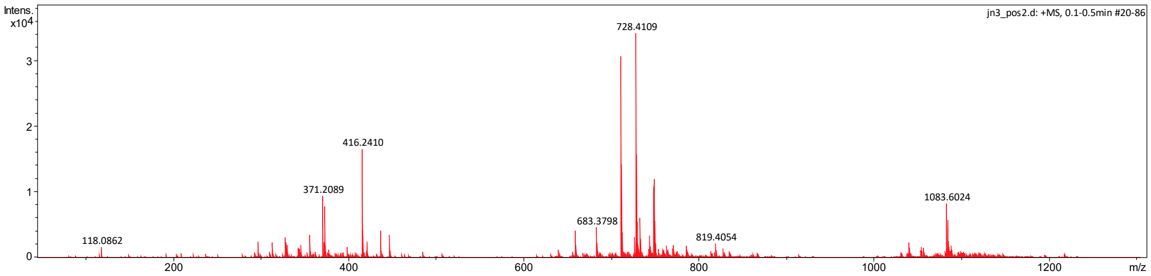

Figure S6. HREIMS spectrum of compound **JN1**.

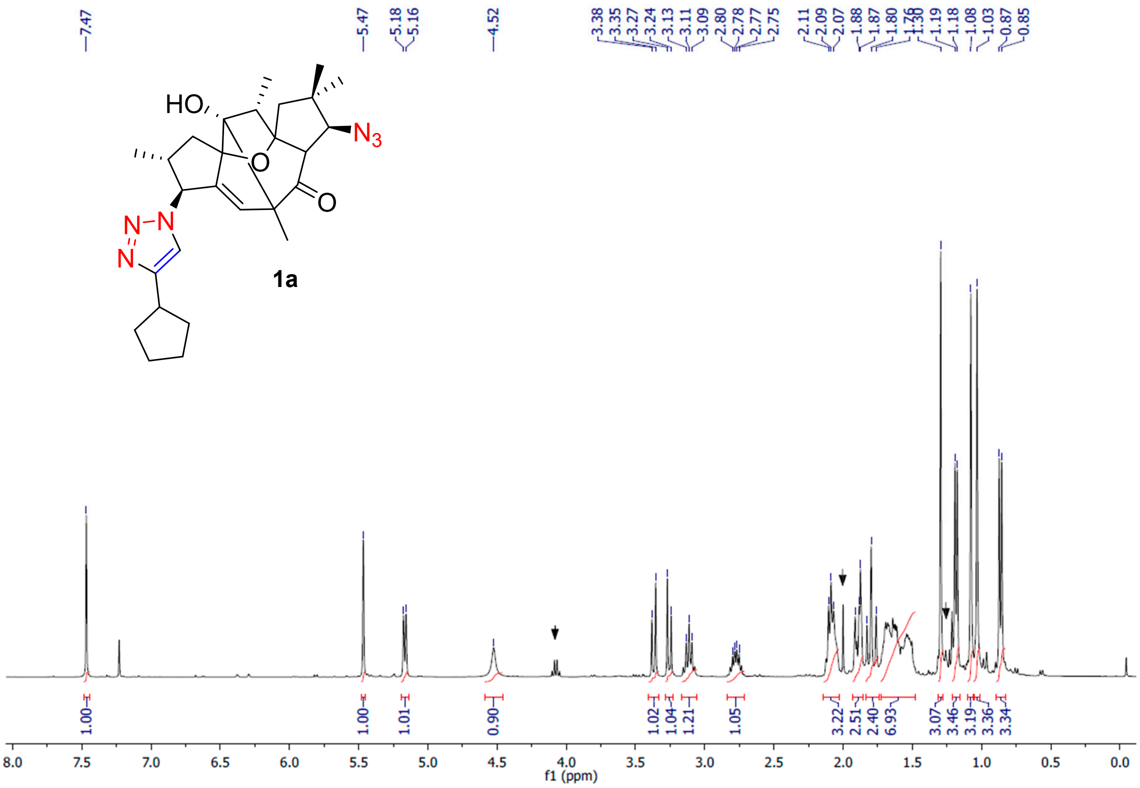

Figure S7. ^{1}H NMR spectra in CDCl3 of compound **1a**.

Note: Residual EtOAc signals from purification are observed (δ ≈ 4.12, 2.05, and 1.25 ppm; black arrows).

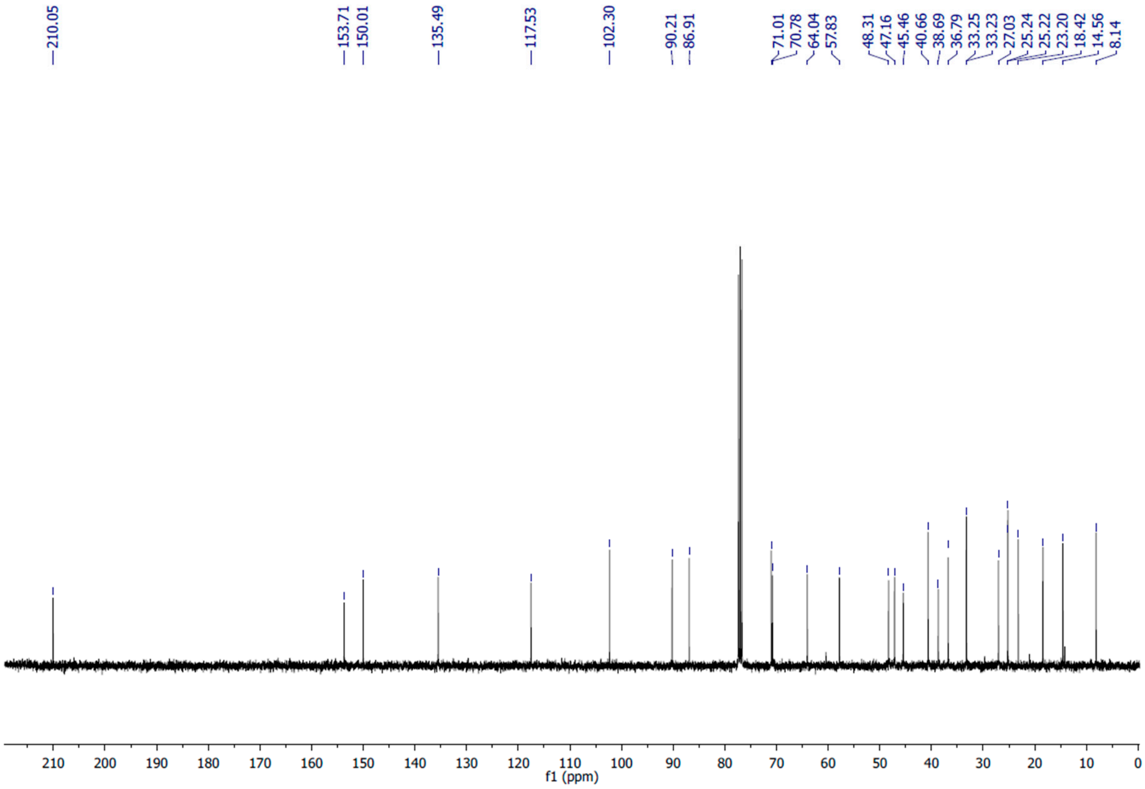

Figure S8. ^{13}C NMR spectra in $CDCl_3$ of compound **1a**.

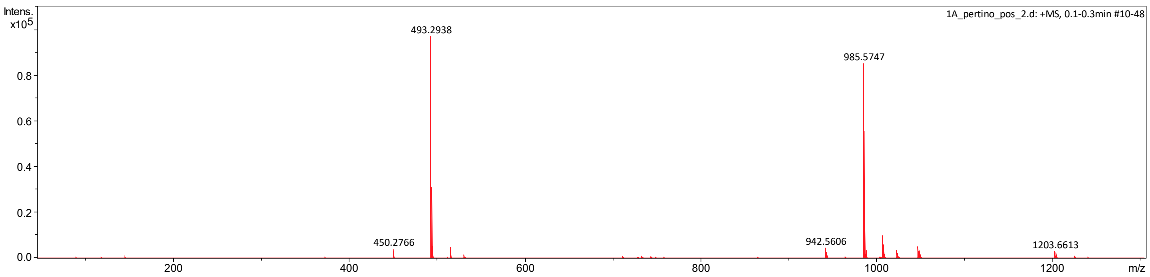

Figure S9. HREIMS spectrum of compound **1a**.

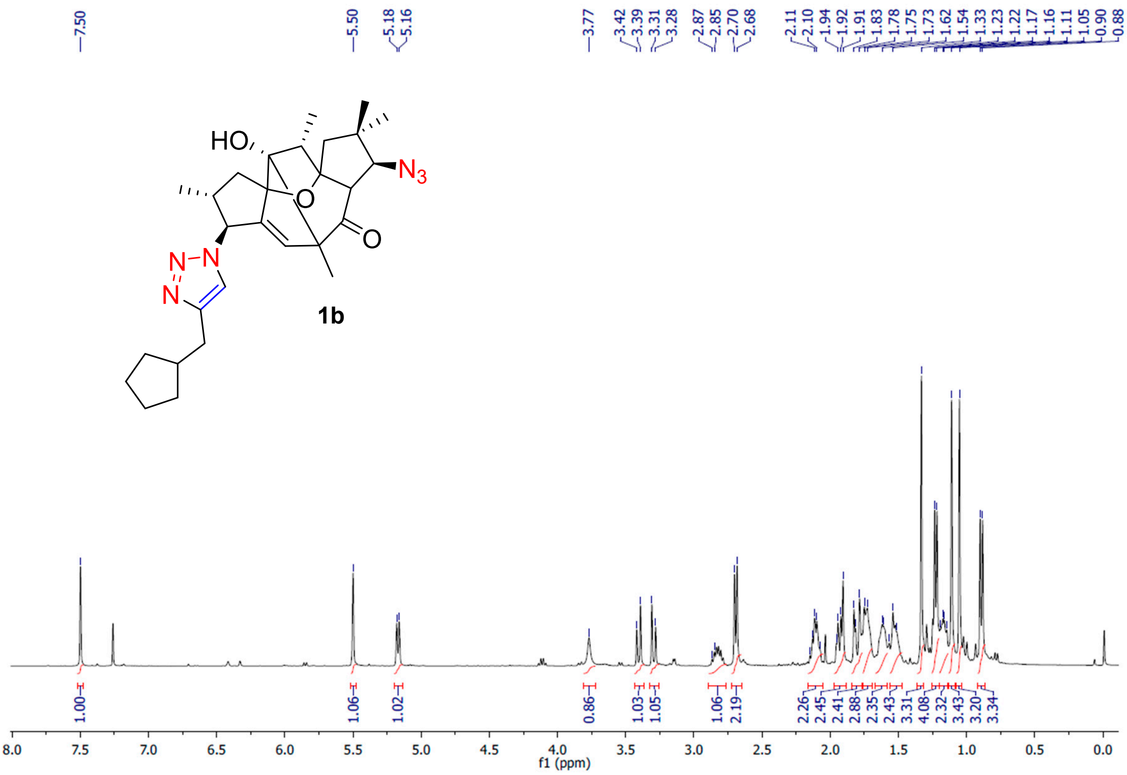

Figure S10. ^{1}H NMR spectra in $CDCl_3$ of compound **1b**.

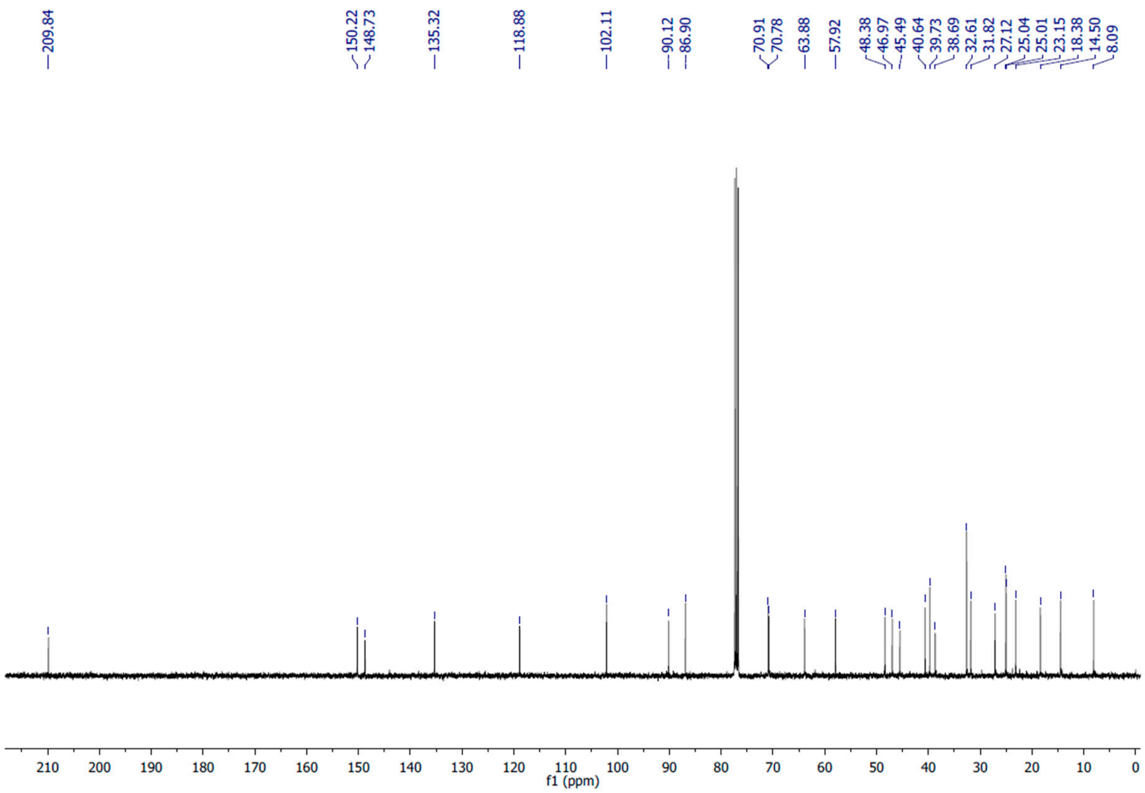

Figure S11. ^{13}C NMR spectra in $CDCl_3$ of compound **1b**.

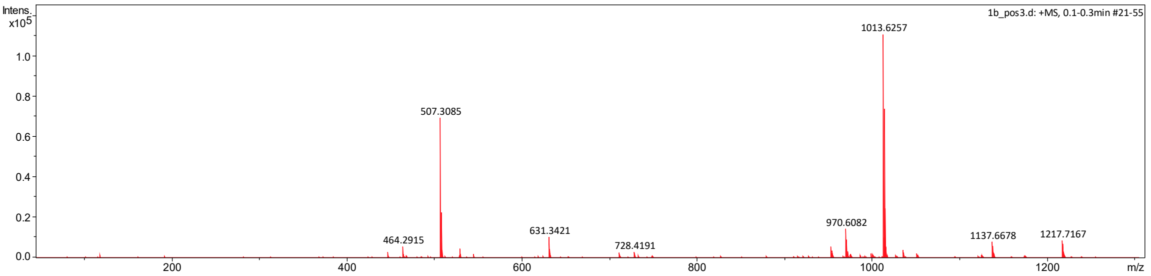

Figure S12. HREIMS spectrum of compound **1b**.

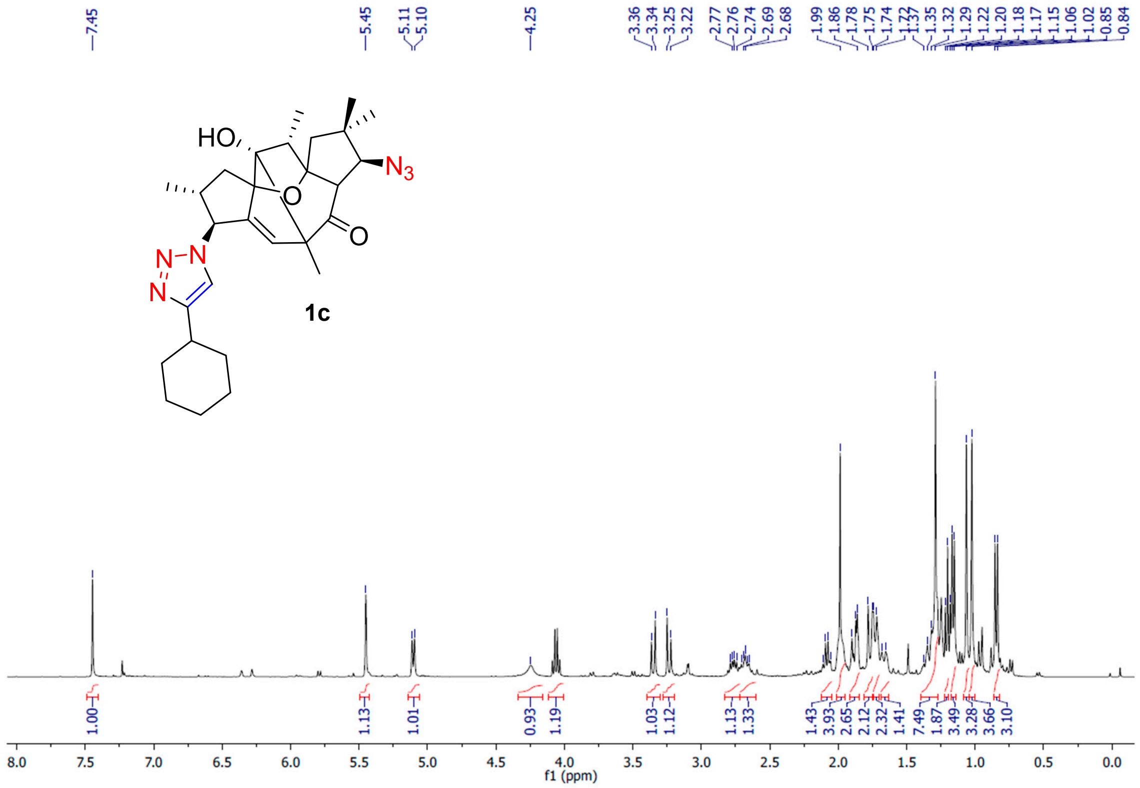

Figure S13. ^{1}H NMR spectra in $CDCl_3$ of compound **1c**.

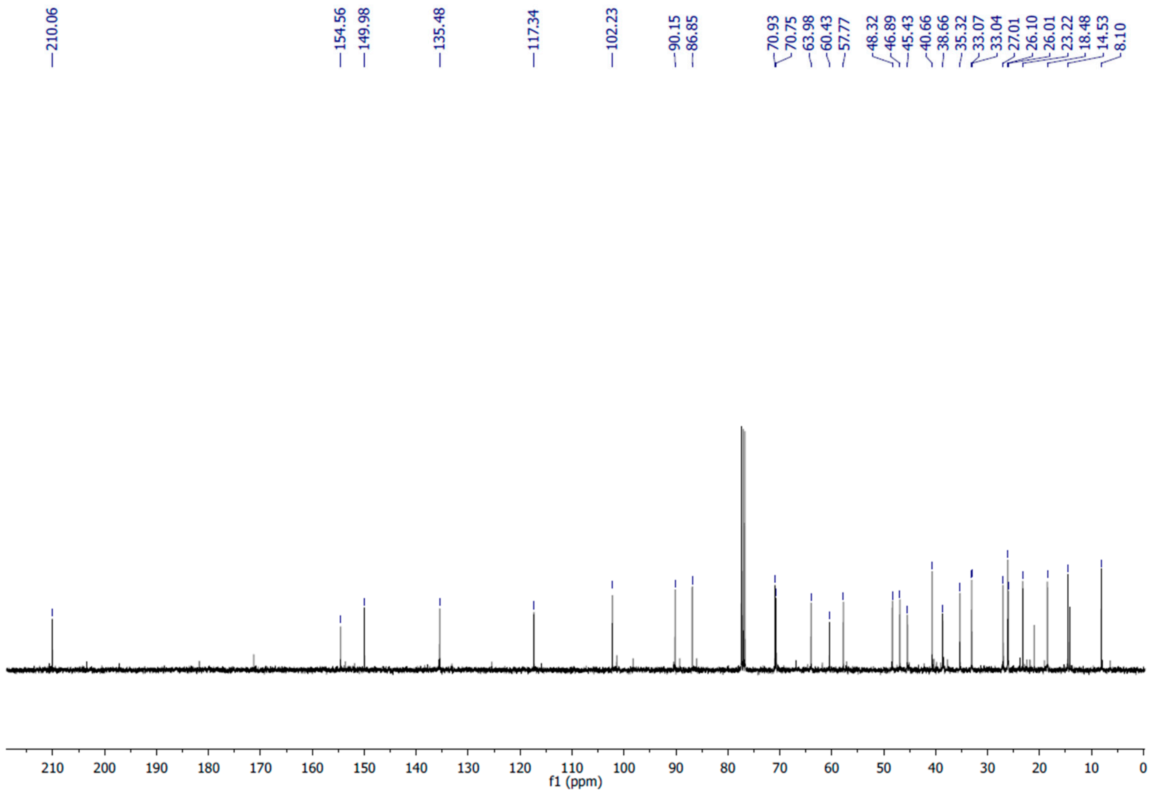

Figure S14. ^{13}C NMR spectra in $CDCl_3$ of compound **1c**.

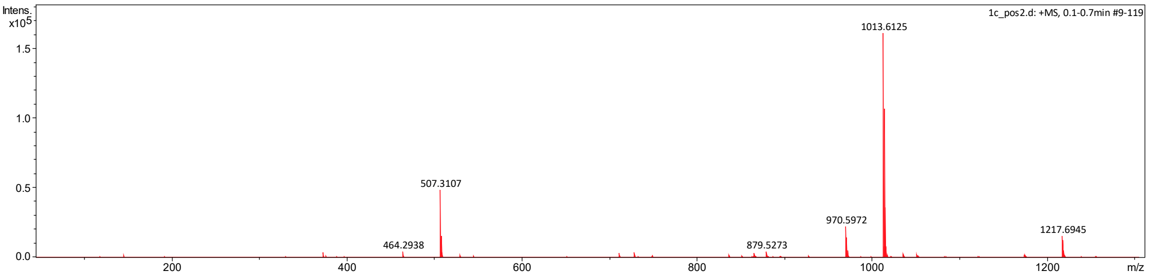

Figure S15. HREIMS spectrum of compound **1c**.

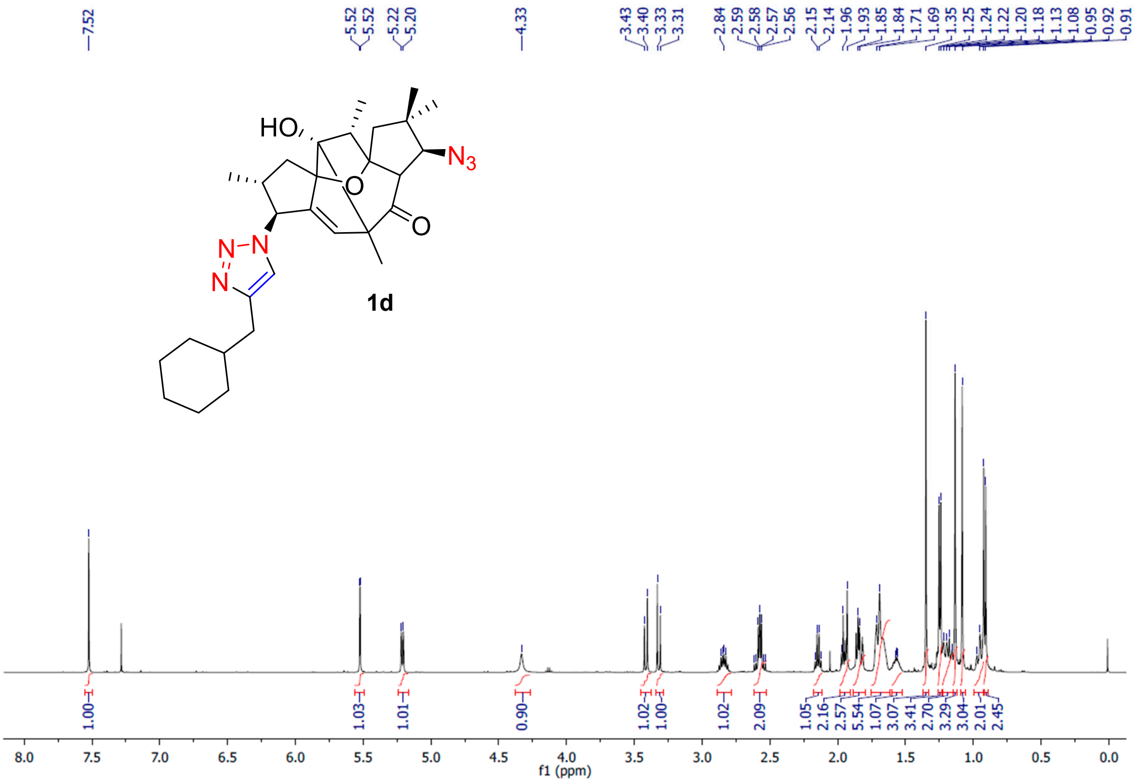

Figure S16. ^{1}H NMR spectra in $CDCl_3$ of compound **1d**.

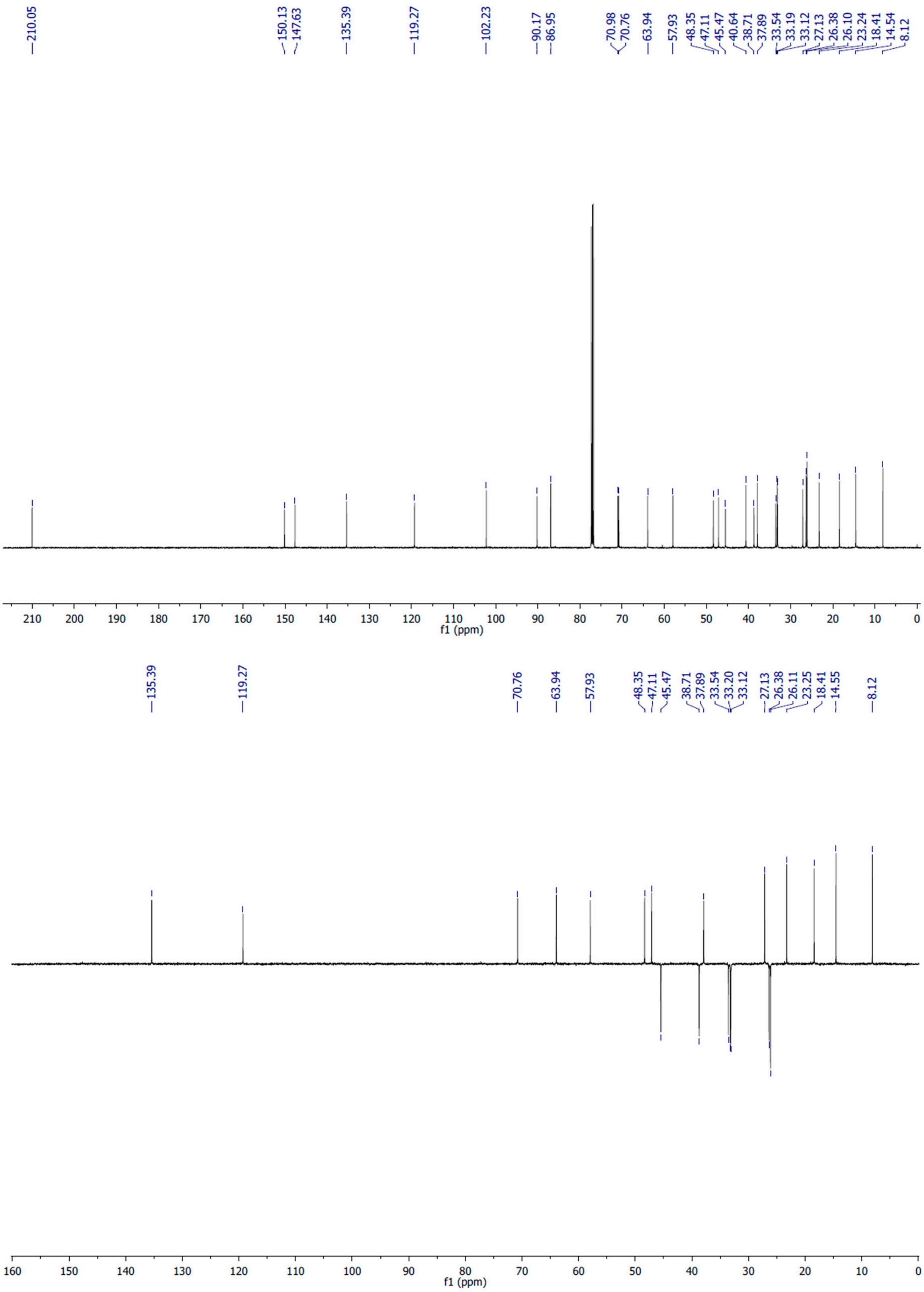

Figure S17. ^{13}C NMR and DEPT-135 spectra in $CDCl_3$ of compound **1d**.

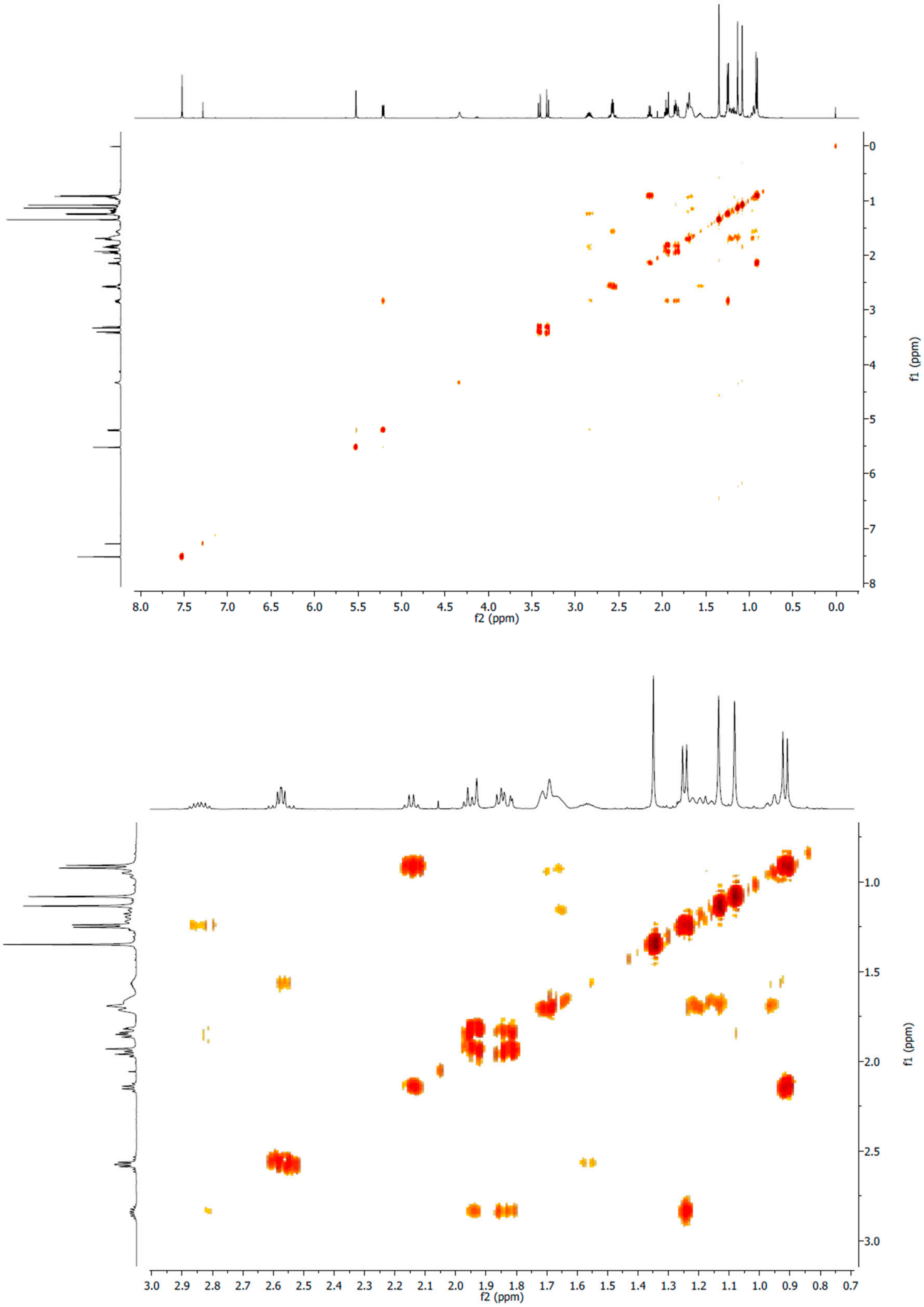

Figure S18. ^{1}H-^{1}H COSY spectra in $CDCl_3$ of compound **1d**.

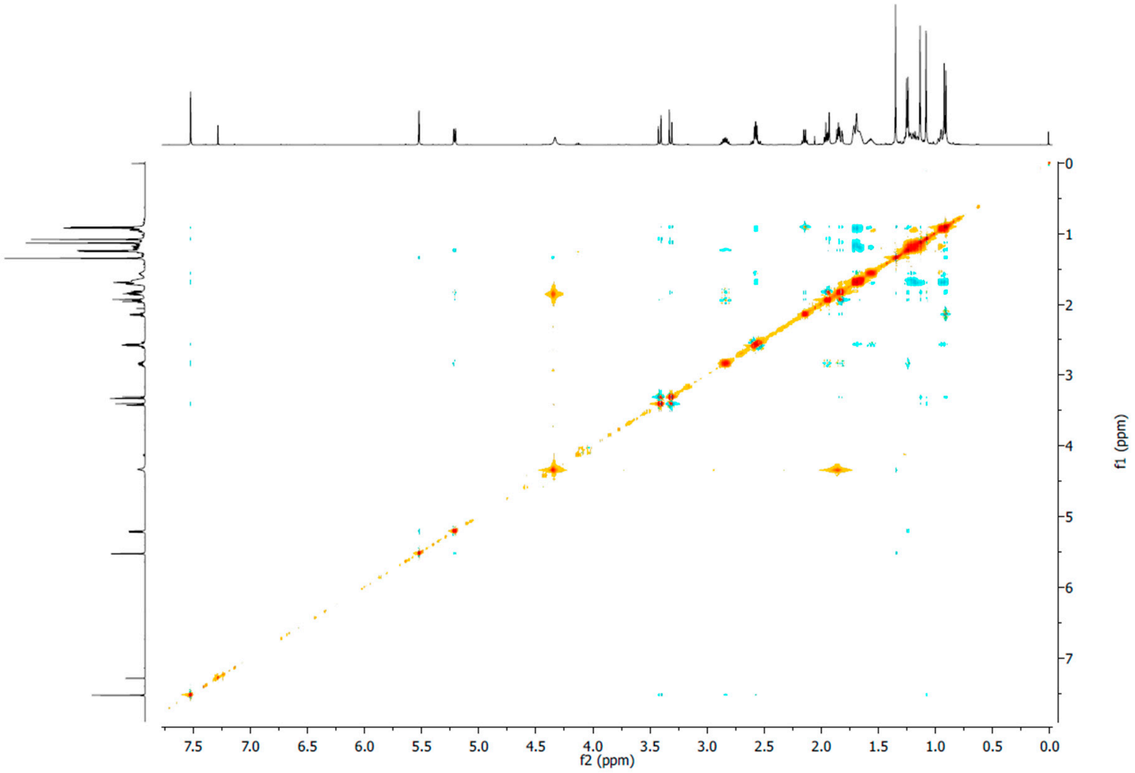

Figure S19. NOESY spectra in $CDCl_3$ of compound **1d**.

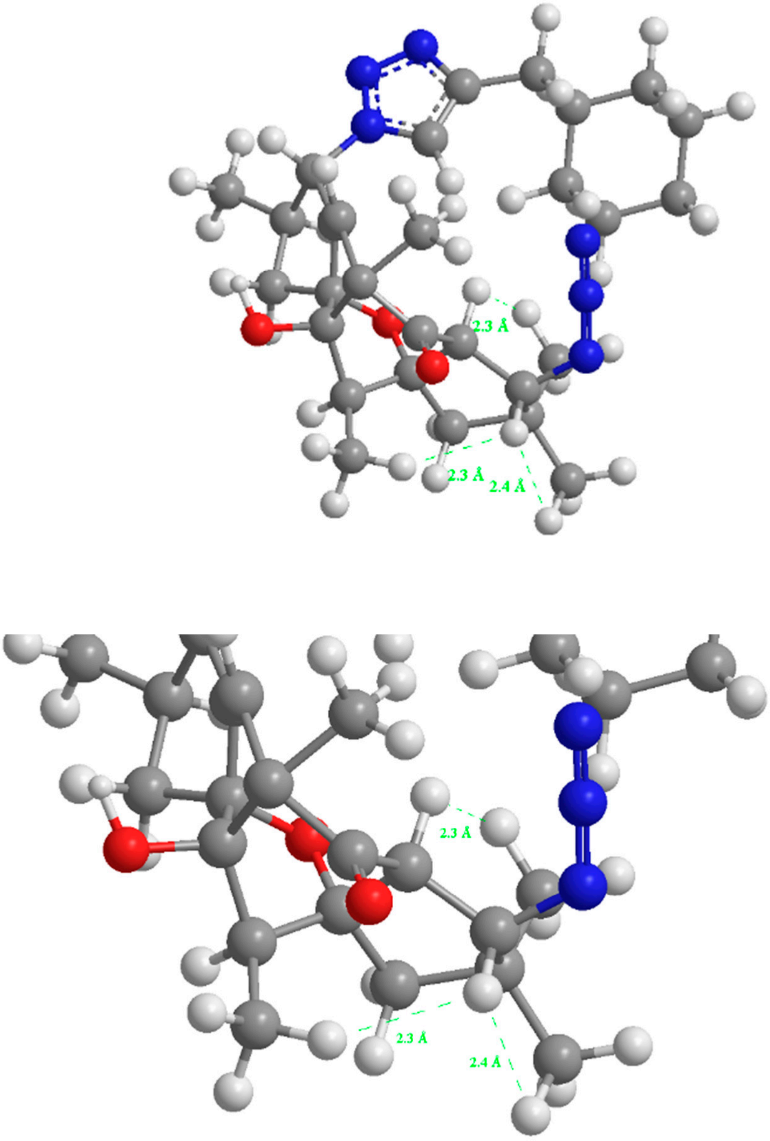

Figure S20. Three-dimensional representation of compound **1d** highlighting the NOESY correlations involving protons H-8 and H-9.

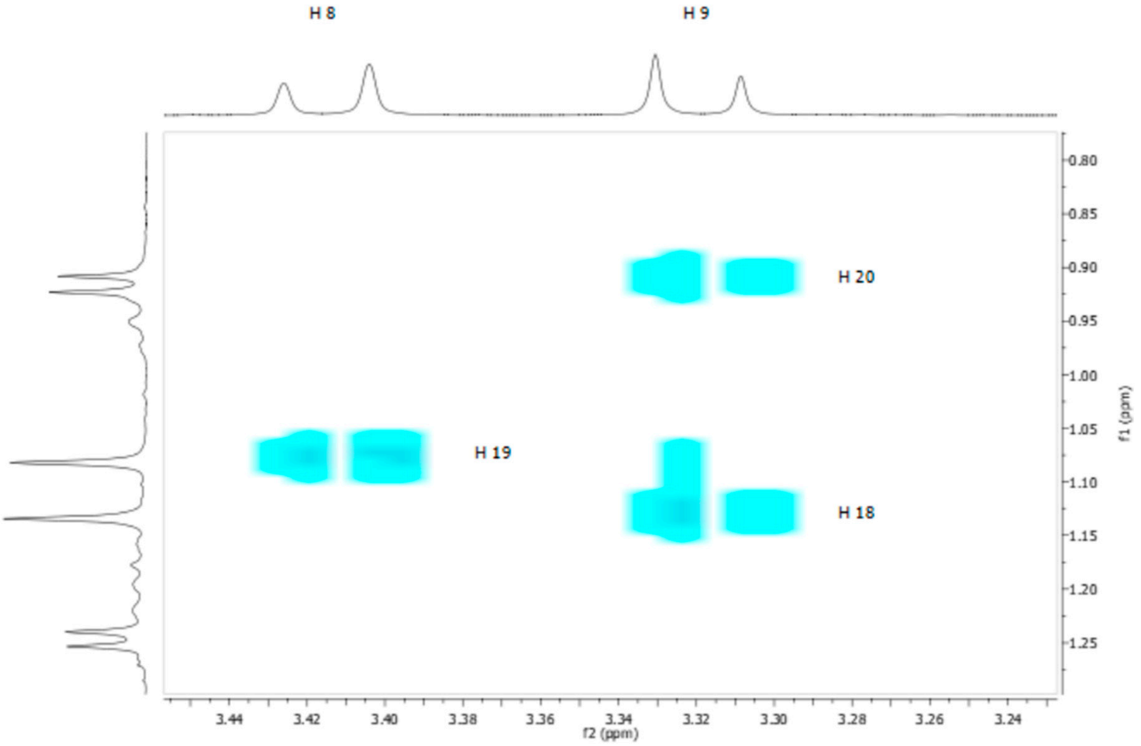

Figure S21. Key NOESY correlations involving protons H-8 and H-9 in compound **1d**.

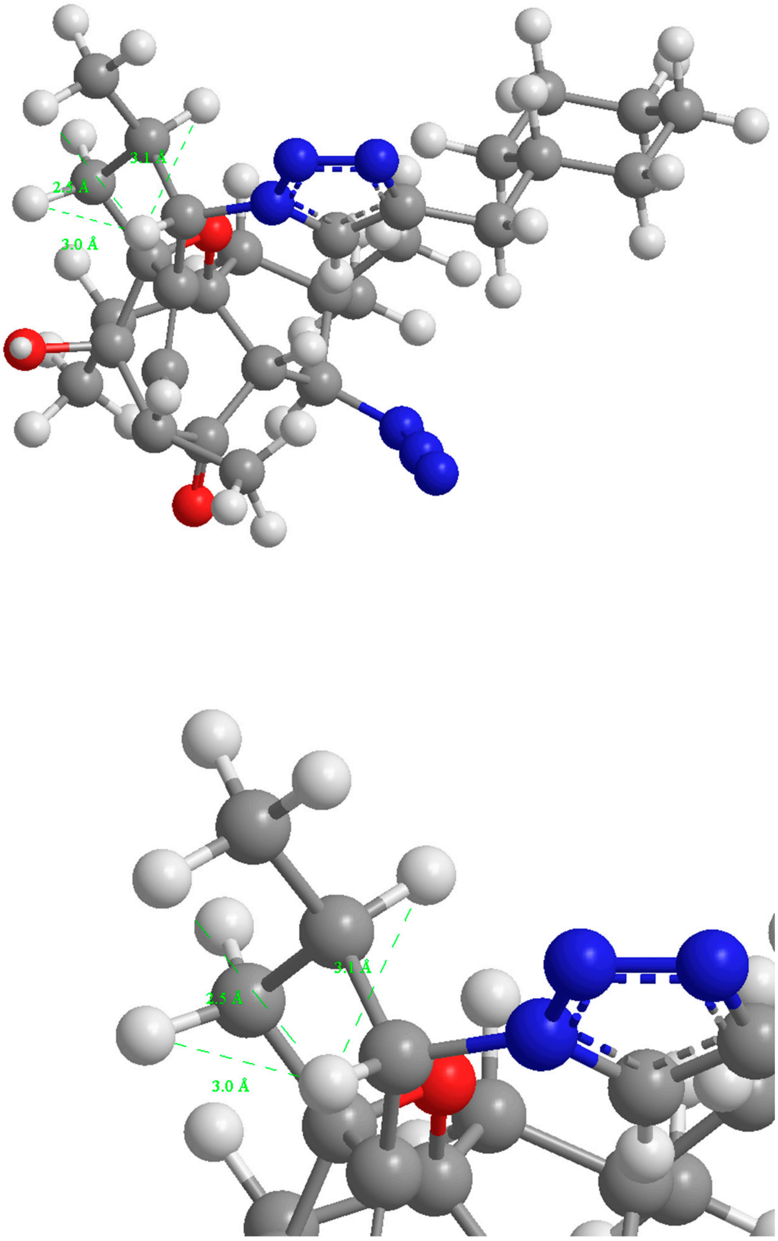

Figure S22. Three-dimensional representation of compound **1d** highlighting the NOESY correlations involving proton H-3.

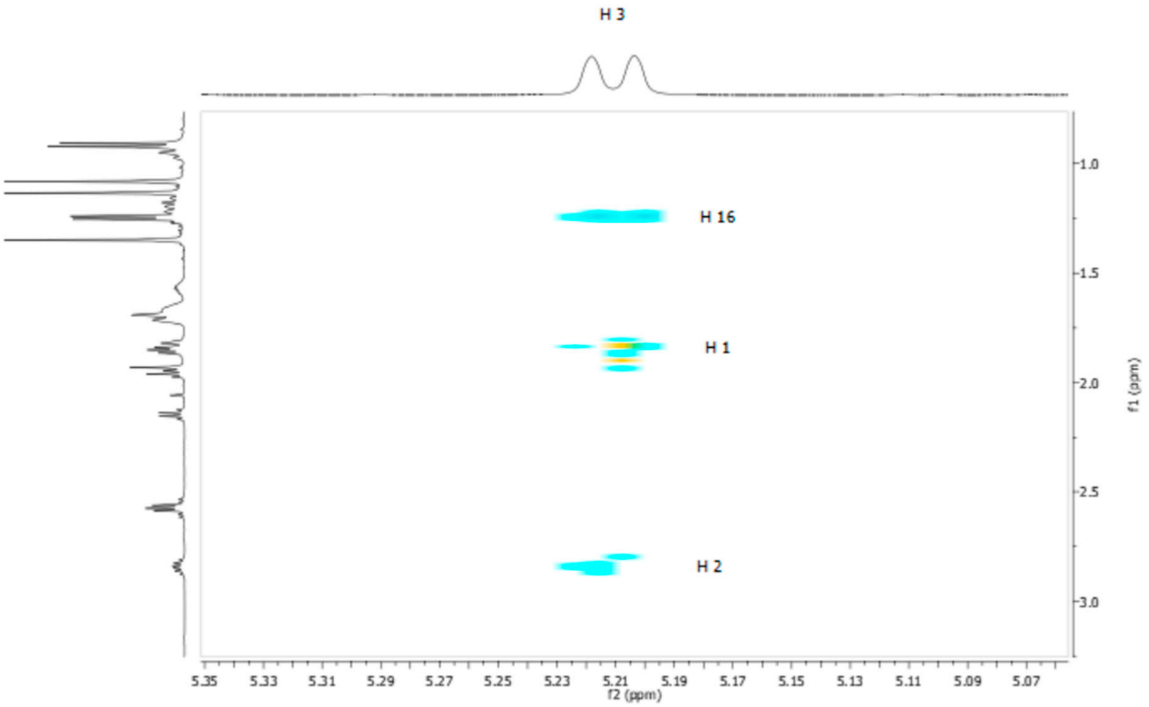

Figure S23. Key NOESY correlations involving proton H-3 in compound **1d**.

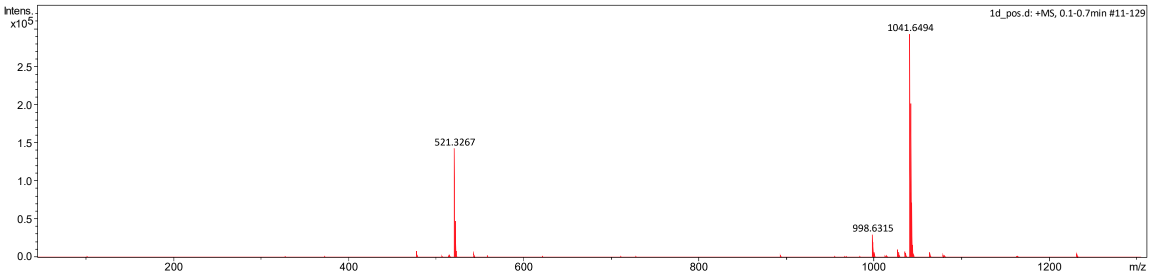

Figure S24. HREIMS spectrum of compound **1d**.

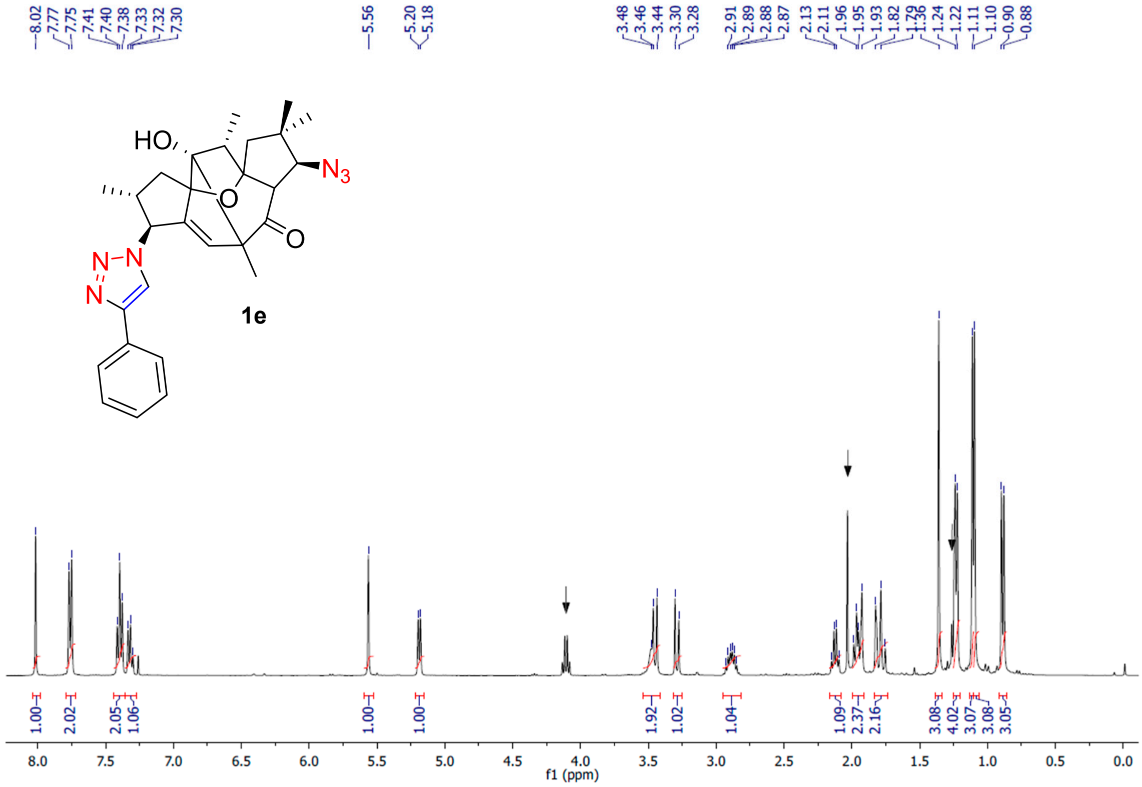

Figure S25. ^{1}H NMR spectra in $CDCl_3$ of compound **1e**.

Note: Residual EtOAc signals from purification are observed (δ ≈ 4.12, 2.05, and 1.25 ppm; black arrows).

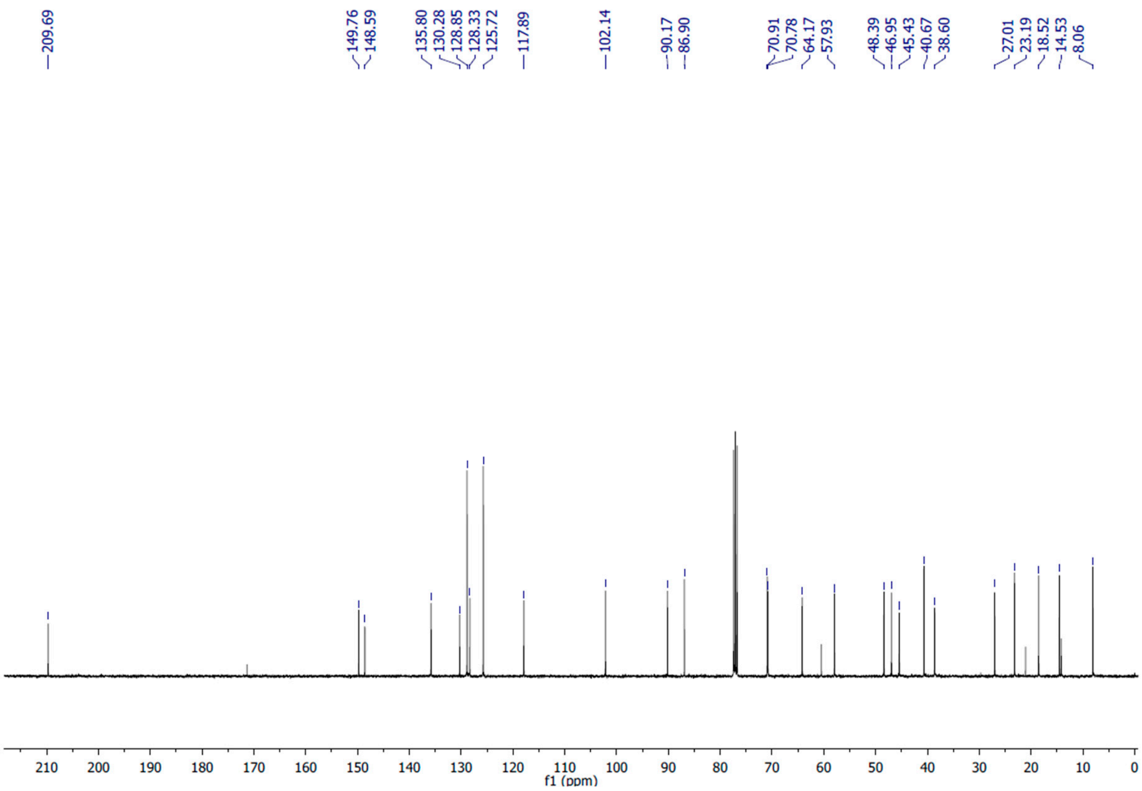

Figure S26. ^{13}C NMR spectra in $CDCl_3$ of compound **1e**.

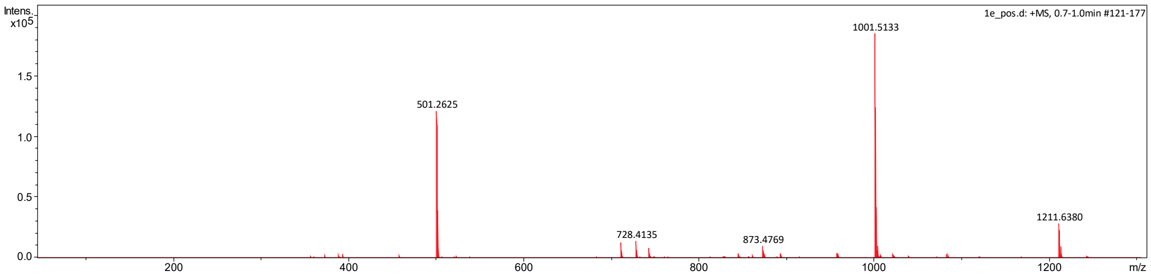

Figure S27. HREIMS spectrum of compound **1e**.

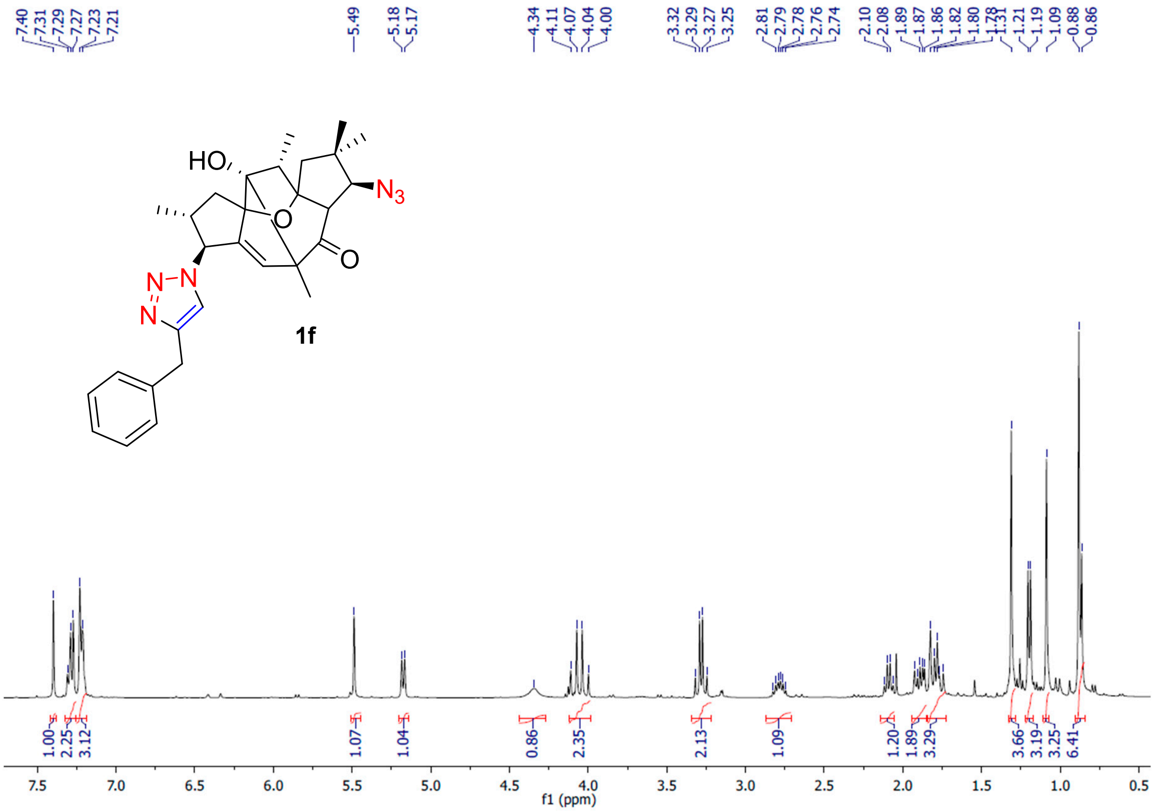

Figure S28. ^{1}H NMR spectra in $CDCl_3$ of compound **1f**.

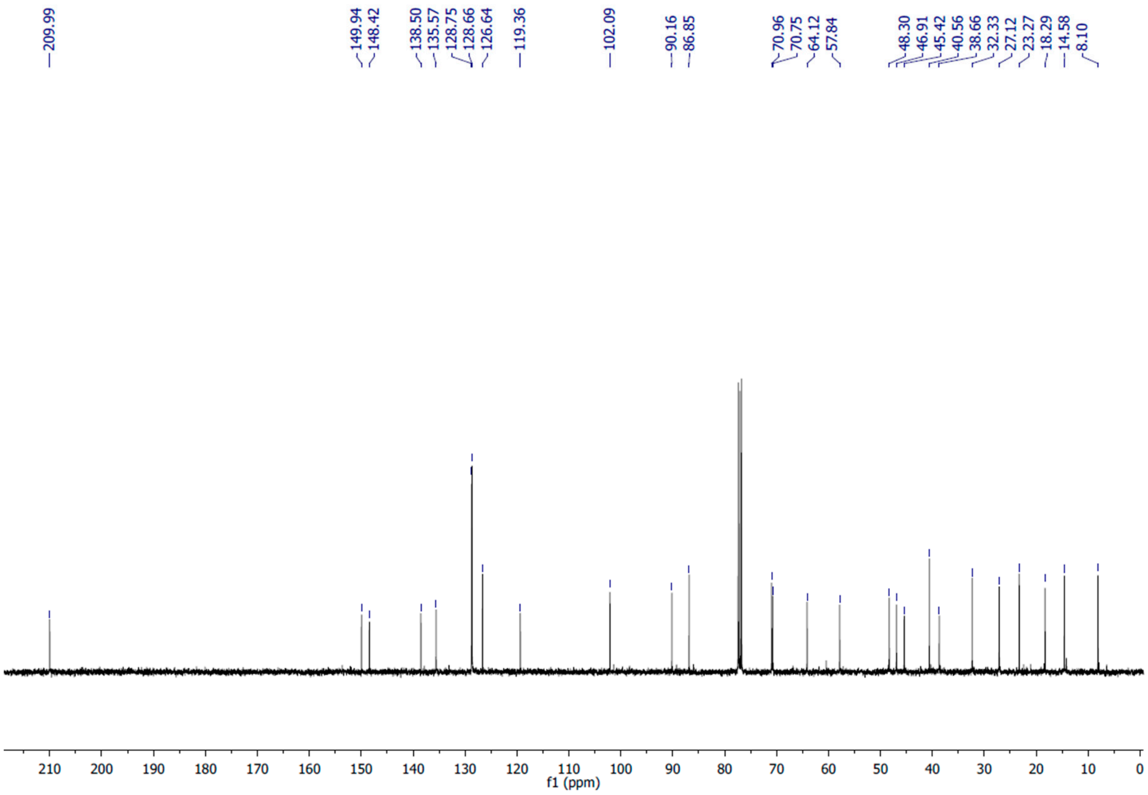

Figure S29. ^{13}C NMR spectra in $CDCl_3$ of compound **1f**.

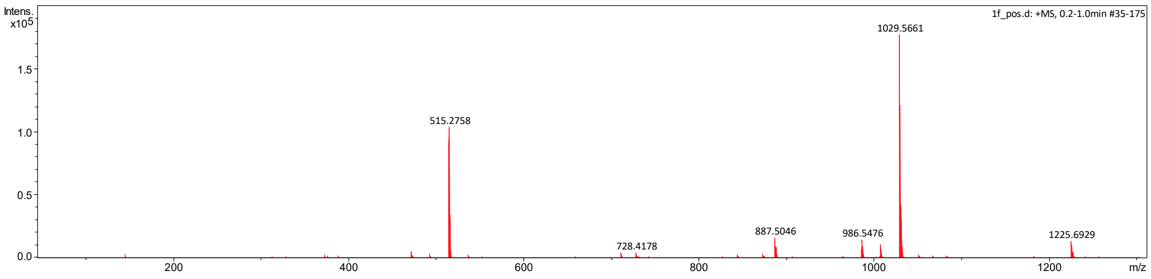

Figure S30. HREIMS spectrum of compound **1f**.

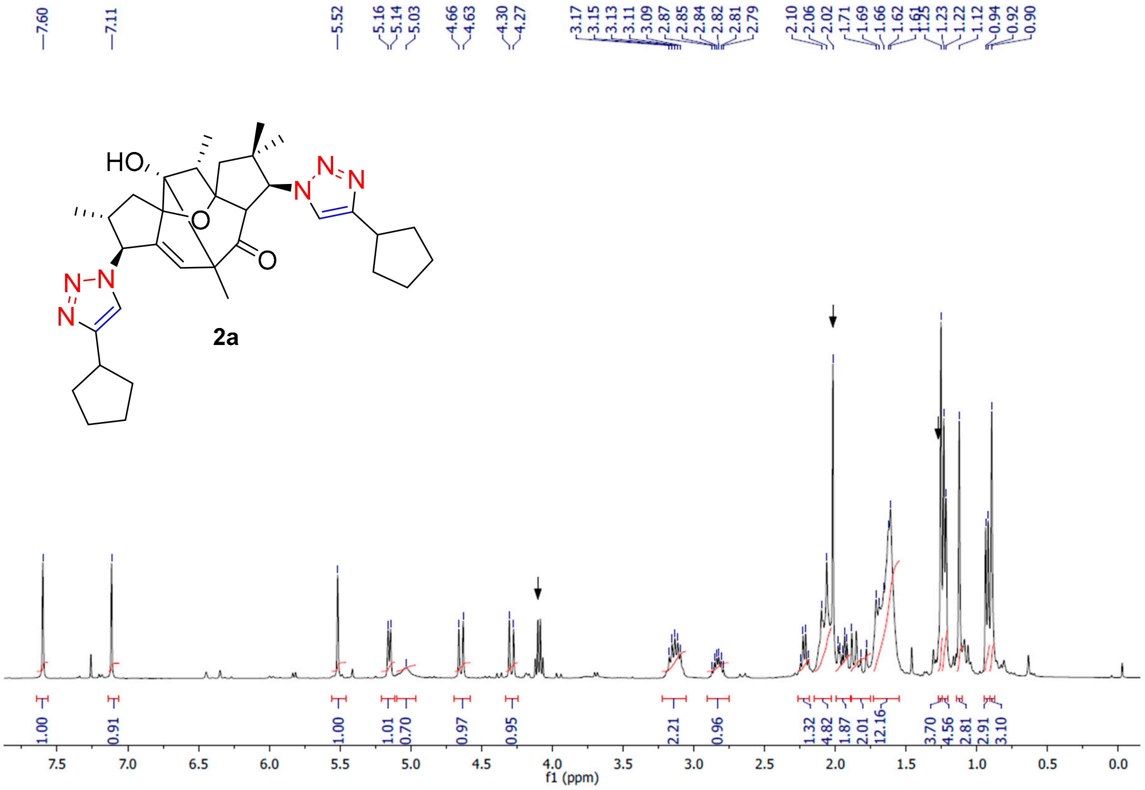

Figure S31. ^{1}H NMR spectra in $CDCl_3$ of compound **2a**.

Note: Residual EtOAc signals from purification are observed (δ ≈ 4.12, 2.05, and 1.25 ppm; black arrows).

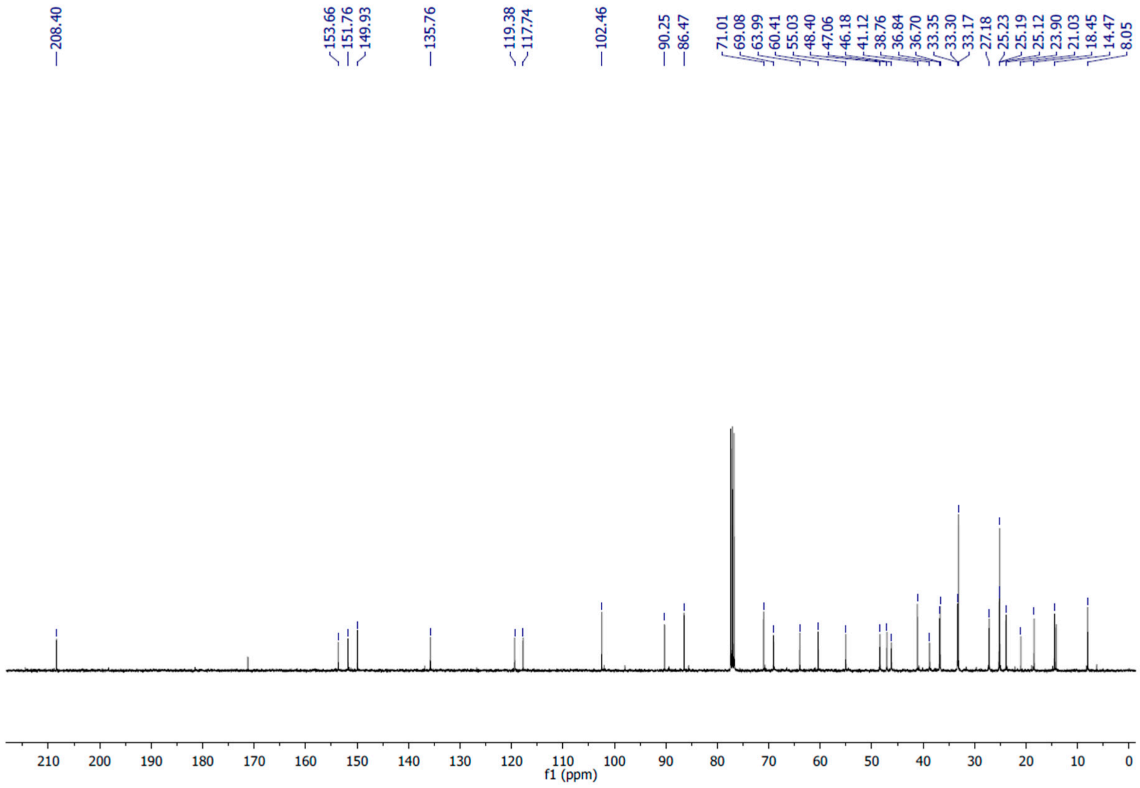

Figure S32 ^{13}C NMR spectra in CDCl3 of compound **2a**.

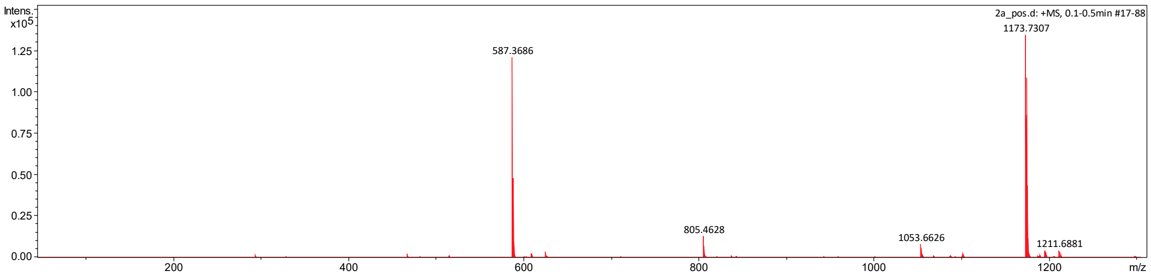

Figure S33. HREIMS spectrum of compound **2a**.

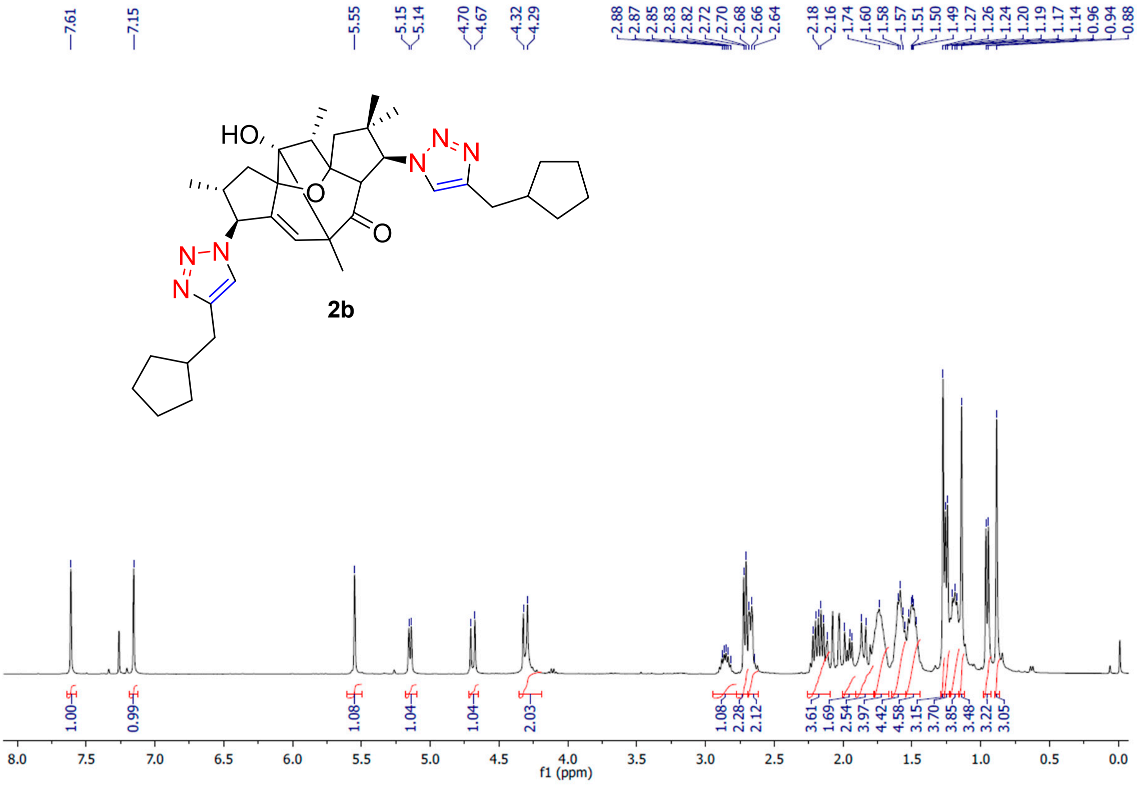

Figure S34. ^{1}H NMR spectra in $CDCl_3$ of compound **2b**.

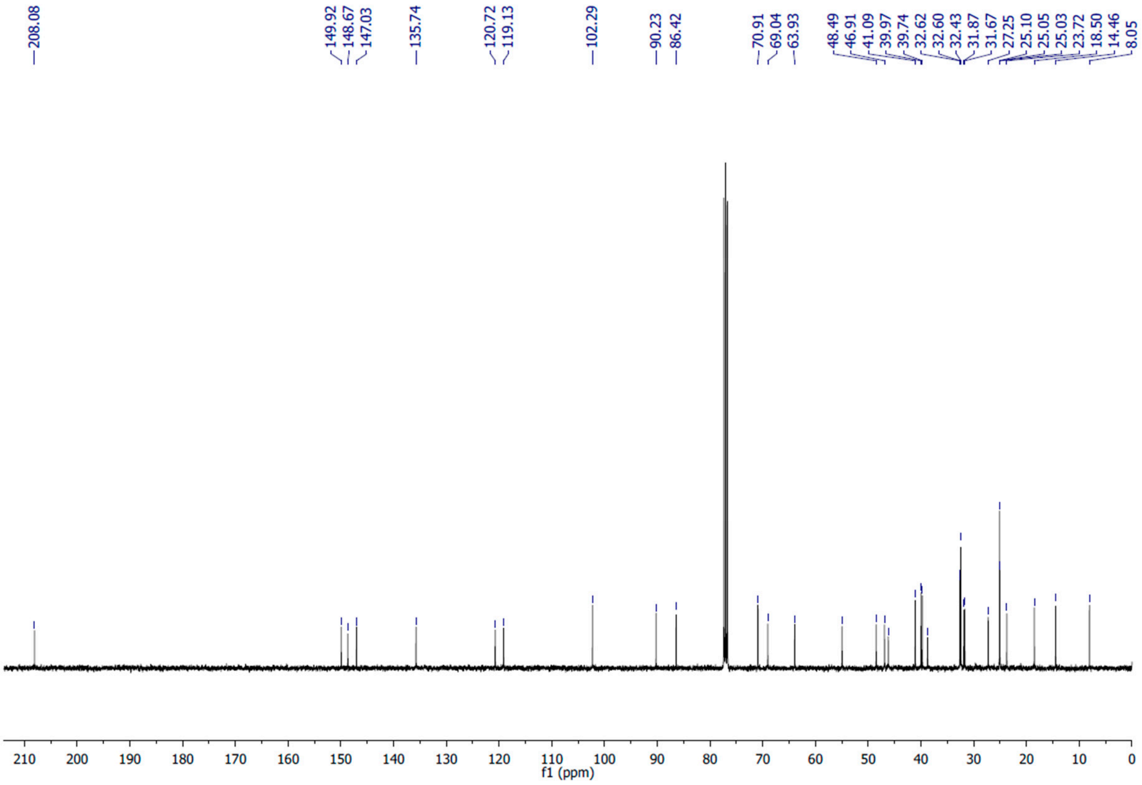

Figure S35. ^{13}C NMR spectra in $CDCl_3$ of compound **2b**.

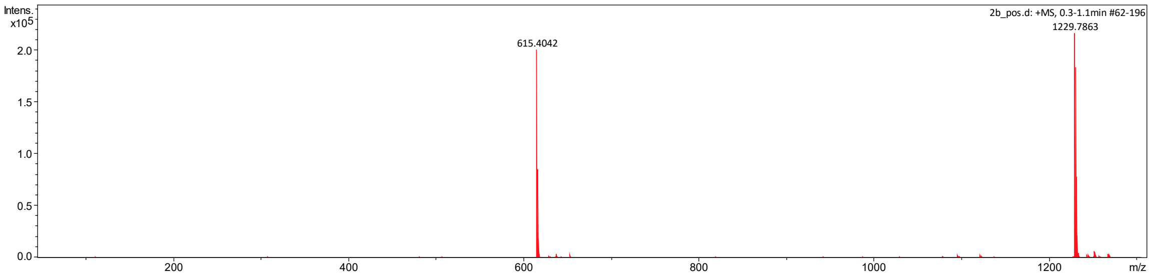

Figure S36 HREIMS spectrum of compound **2b**.

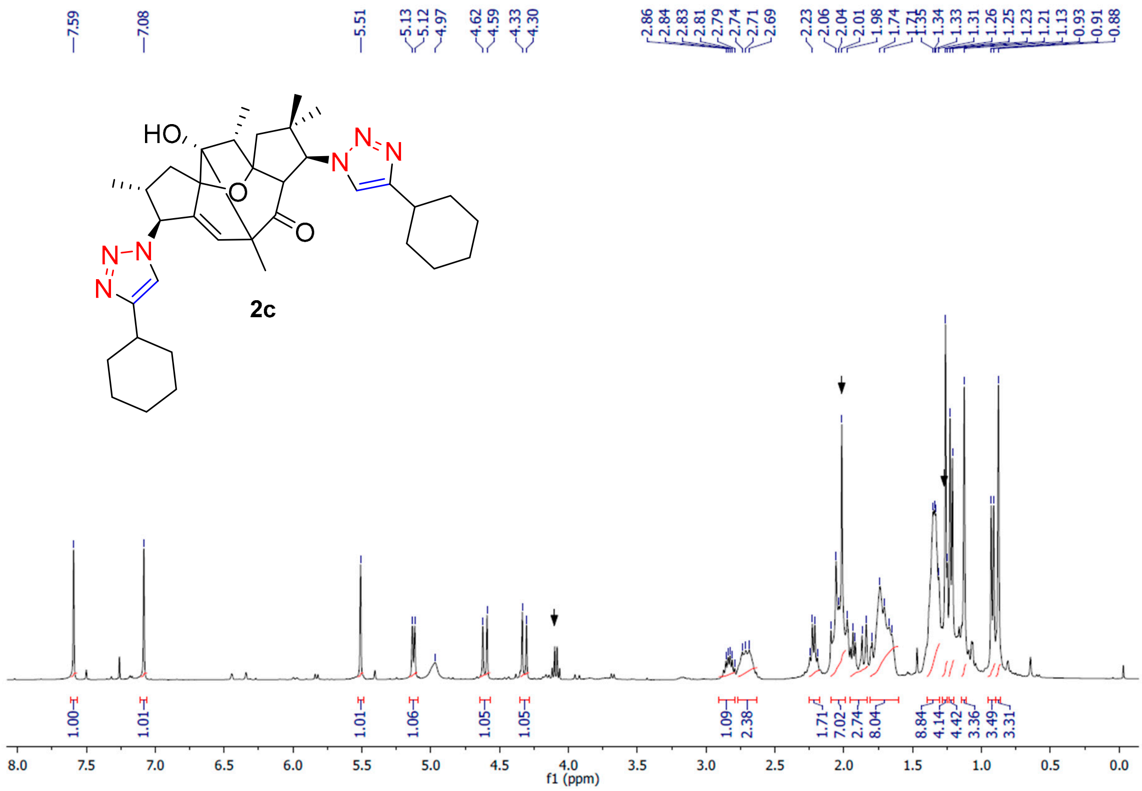

Figure S37. ^{1}H NMR spectra in $CDCl_3$ of compound **2c**.

Note: Residual EtOAc signals from purification are observed (δ ≈ 4.12, 2.05, and 1.25 ppm; black arrows).

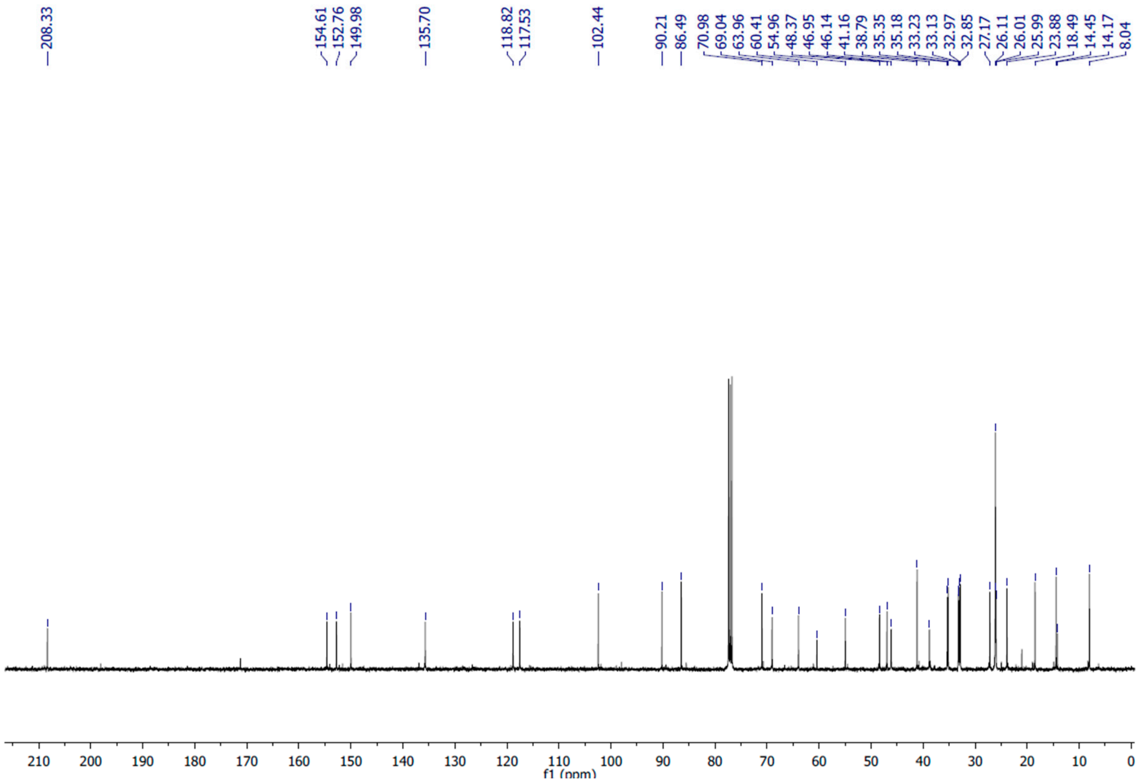

Figure S38 ^{13}C NMR spectra in $CDCl_3$ of compound **2c**.

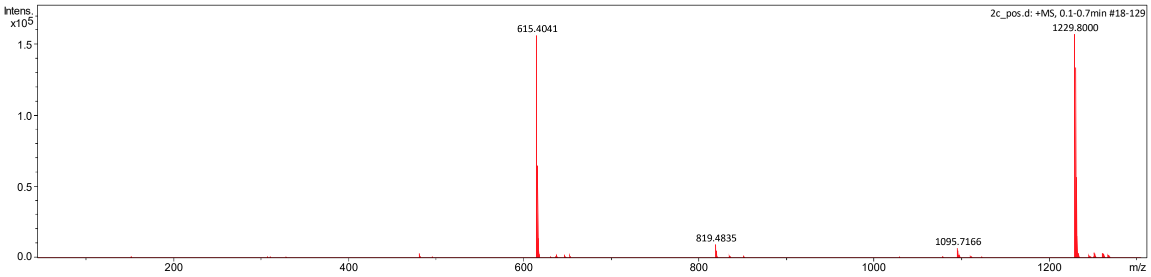

Figure S39. HREIMS spectrum of compound **2c**.

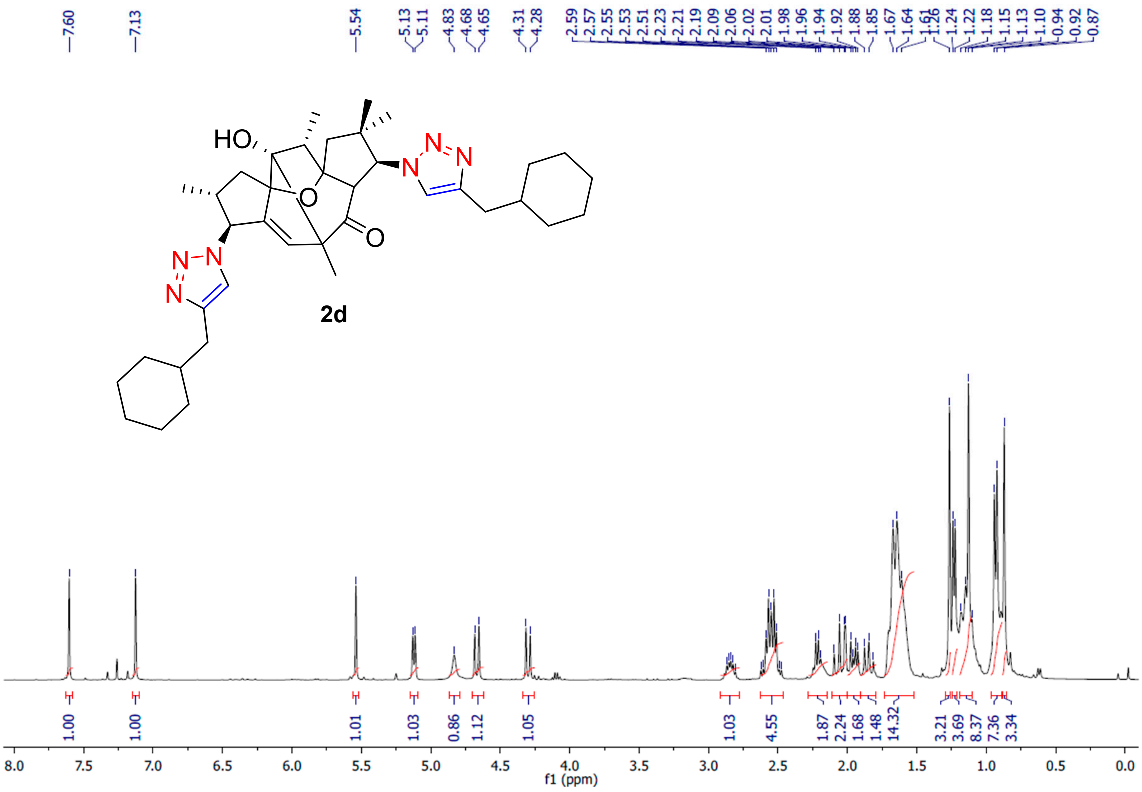

Figure S40. ^{1}H NMR spectra in $CDCl_3$ of compound **2d**.

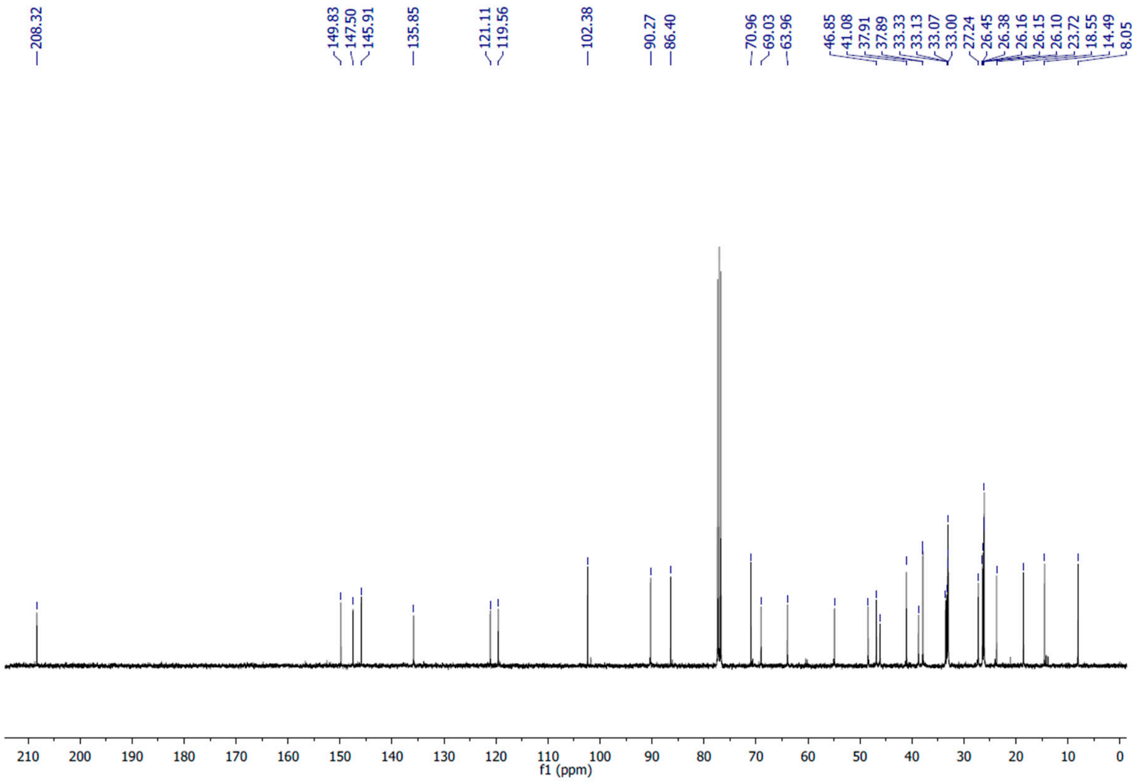

Figure S41. ^{13}C NMR spectra in $CDCl_3$ of compound **2d**.

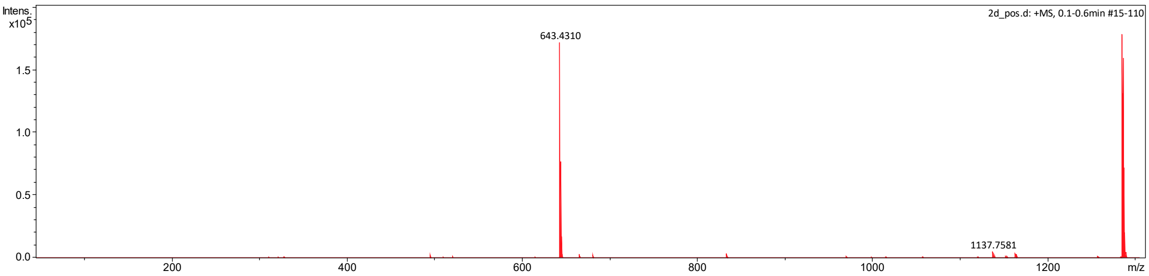

Figure S42. HREIMS spectrum of compound **2d**.

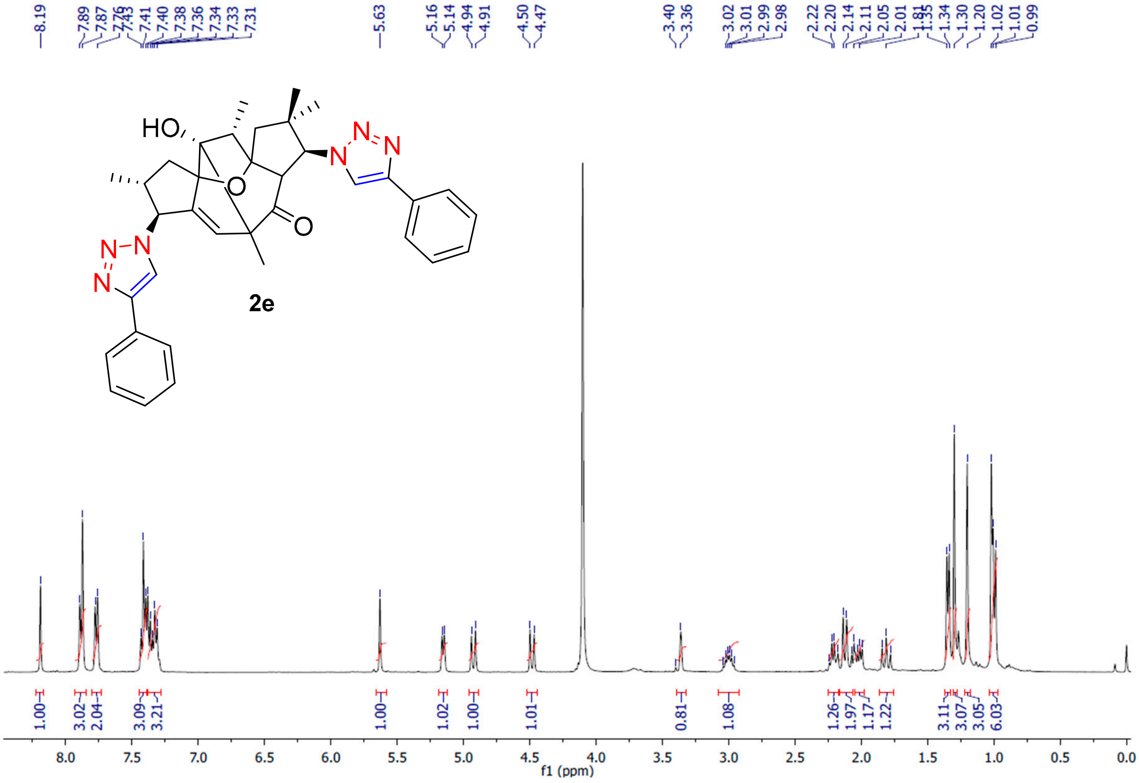

Figure S43. ^{1}H NMR spectra in $CDCl_3/CD_3OD$ of compound **2e**.

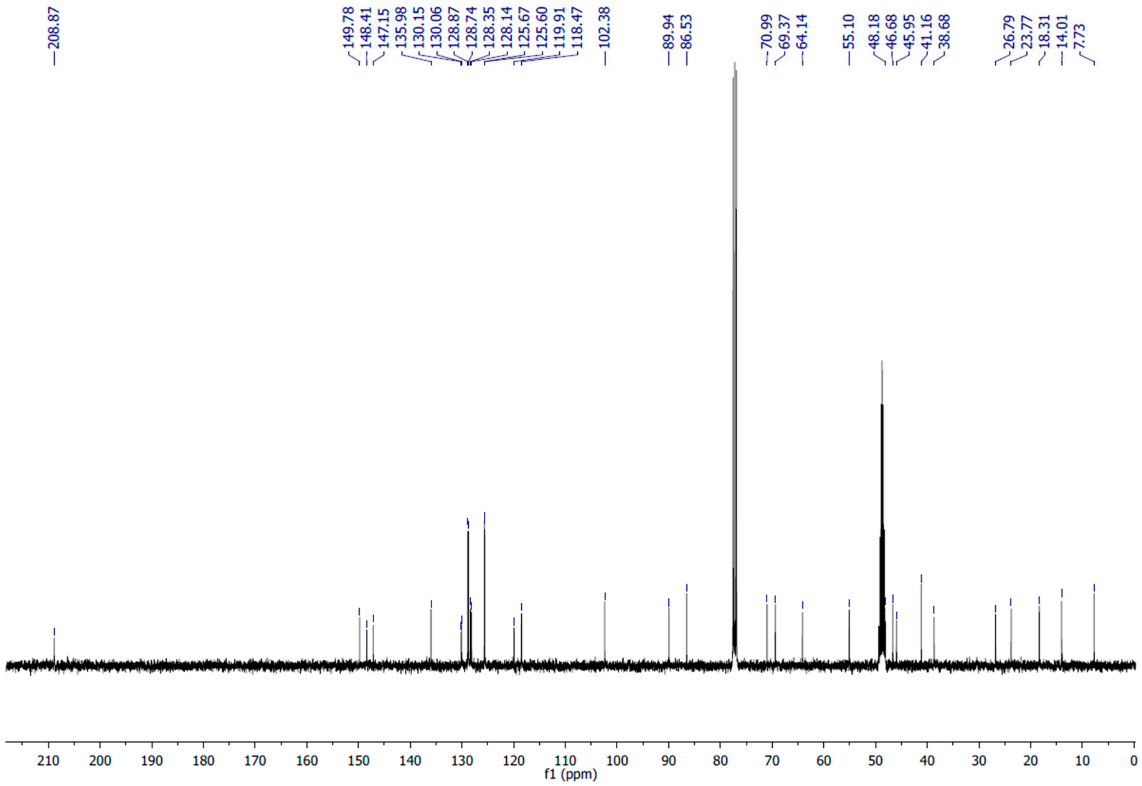

Figure S44. ^{13}C NMR spectra in $CDCl_3/CD_3OD$ of compound **2e**.

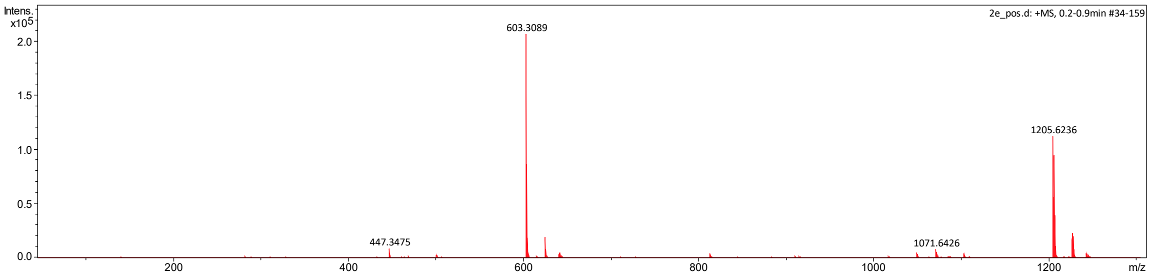

Figure S45. HREIMS spectrum of compound **2e**.

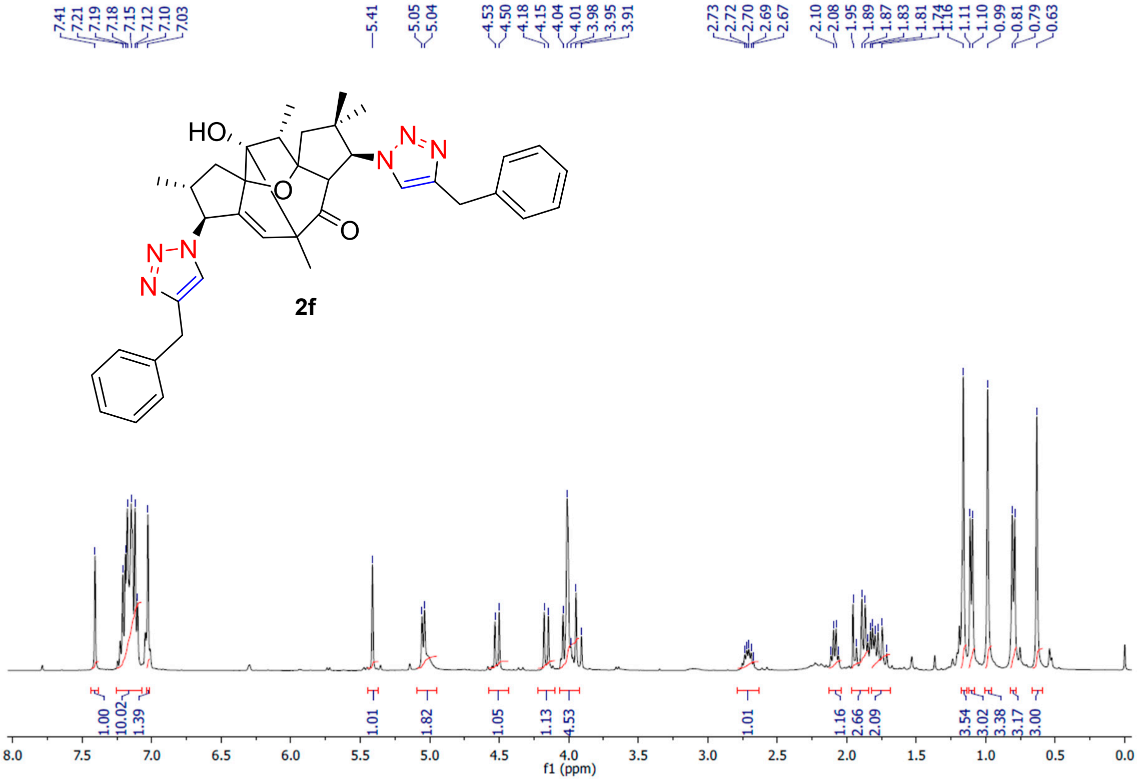

Figure S46. ^{1}H NMR spectra in $CDCl_3$ of compound **2f**.

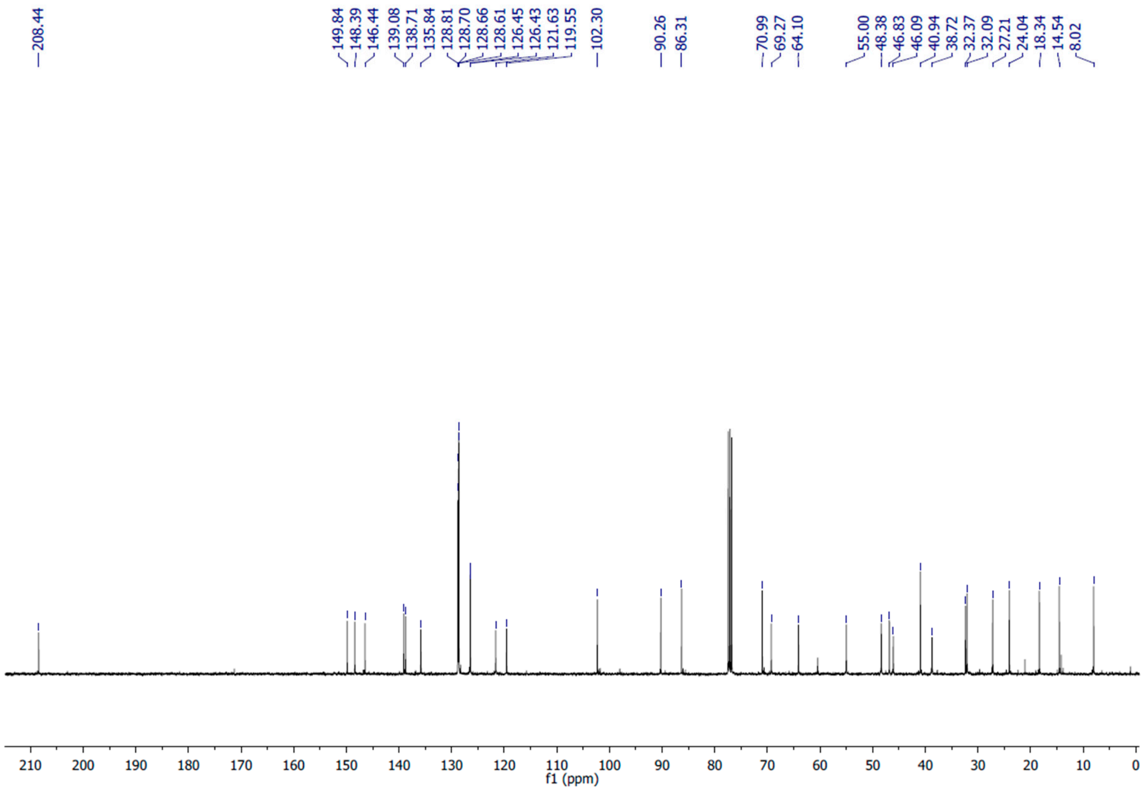

Figure S47. ^{13}C NMR spectra in $CDCl_3$ of compound **2f**.

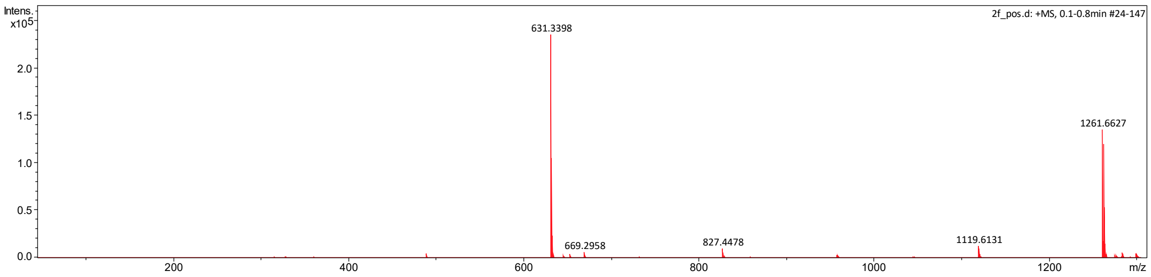

Figure S48. HREIMS spectrum of compound **2f**.

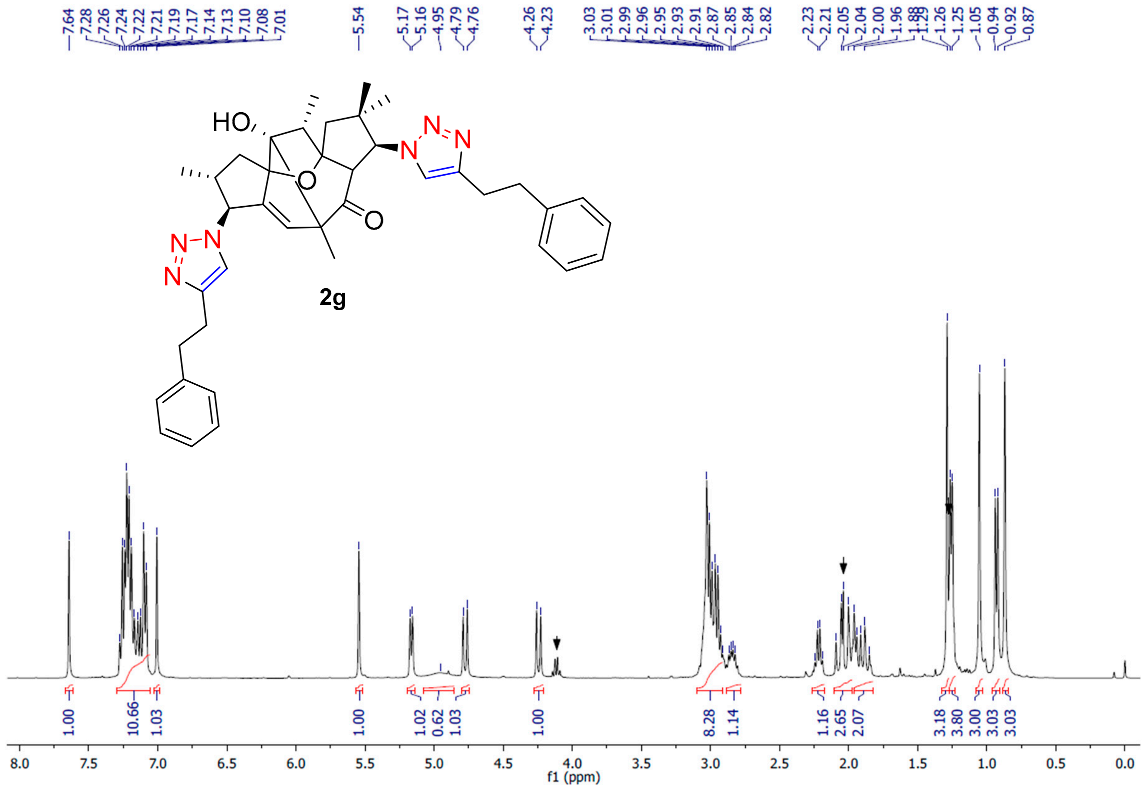

Figure S49. ^{1}H NMR spectra in $CDCl_3$ of compound **2g**.

Note: Residual EtOAc signals from purification are observed (δ ≈ 4.12, 2.05, and 1.25 ppm; black arrows).

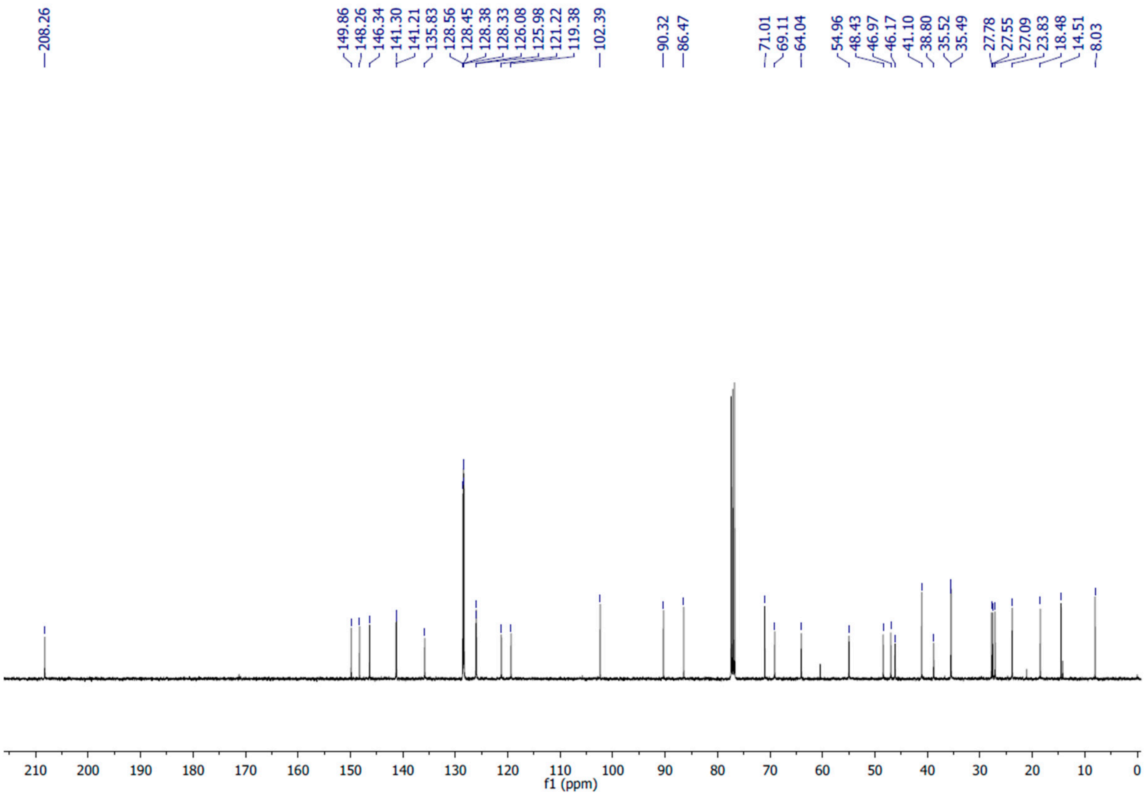

Figure S50. ^{13}C NMR spectra in $CDCl_3$ of compound **2g**.

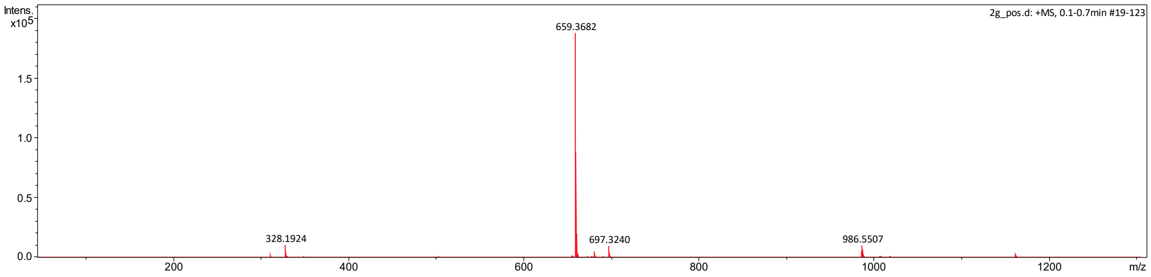

Figure S51. HREIMS spectrum of compound **2g**.

Table S4. Physicochemical and pharmacokinetic descriptors calculated with SwissADME.

Compound	MW[1]	RB[2]	HBA[3]	HBD[4]	TPSA[5]	Log $P_{o}/_{p}$[6]	Water solubility[7] (mol/L)	GI abs.[8]	BBB[9]	log Kp[10]
**JN1**	398.46	2	9	1	146.03	2.44	4.08×10^{-5}	Low	No	−6.24
**1a**	492.61	3	8	1	126.99	3.31	7.86×10^{-6}	Low	No	−6.71
**1b**	506.64	3	8	1	126.99	3.54	2.26×10^{-6}	Low	No	−6.20
**1c**	506.64	3	8	1	126.99	3.60	2.96×10^{-6}	Low	No	−6.41
**1d**	520.67	3	8	1	126.99	3.55	8.50×10^{-7}	Low	No	−5.90
**1e**	500.59	3	8	1	126.99	3.33	5.35×10^{-6}	Low	No	−6.76
**1f**	514.62	4	8	1	126.99	3.46	3.34×10^{-6}	Low	No	−6.53
**2a**	586.77	4	7	1	107.95	4.05	1.64×10^{-6}	Low	No	−7.17
**2b**	614.82	5	7	1	107.95	4.81	1.34×10^{-7}	Low	No	−5.16
**2c**	614.82	5	7	1	107.95	4.65	2.33×10^{-7}	Low	No	−6.63
**2d**	642.87	5	7	1	107.95	5.36	1.91×10^{-8}	Low	No	−5.56
**2e**	602.73	4	7	1	107.95	4.07	8.41×10^{-7}	Low	No	−7.27
**2f**	630.78	6	7	1	107.95	4.42	3.36×10^{-7}	High	No	−7.02
**2g**	658.83	6	7	1	107.95	4.99	1.34×10^{-7}	Low	No	−6.78

[1] Molecular weight (g/mol); [2] number of rotatable bonds; [3] number of hydrogen bond acceptors; [4] number of hydrogen bond donors; [5] topological polar surface area ($Å^2$); [6] consensus Log Po/w (average of iLOGP, XLOGP3, WLOGP, MLOGP, and SILICOS-IT predictions); [7] ESOL-predicted water solubility; [8] gastrointestinal absorption; [9] blood–brain barrier permeation; [10] skin permeation coefficient predicted by the QSPR model (cm/s).

Table S5. Systematic IUPAC names and SMILES of synthesized compounds

Compound	IUPAC Name	SMILES
**JN1**	(1*S*,2*R*,3b*R*,4*S*,7*S*)-1,7-diazido-3b-hydroxy-2,4,6,6,8a-pentamethyl-1,2,3,3b,4,5,6,7,7a,8a-decahydro-8H-3a,4a-epoxycyclopenta[a]-*s*-indacen-8-one	O=C(C1[C@H](N=[N+]=[N-])C(C)(C)C2)C3(C)C=C4[C@@H](N=[N+]=[N-])[C@H](C)CC45[C@](O)3[C@H](C)C12O5
**1a**	(1*S*,2*R*,3b*R*,4*S*,7*S*)-7-azido-1-(4-cyclopentyl-1H-1,2,3-triazol-1-yl)-3b-hydroxy-2,4,6,6,8a-pentamethyl-1,2,3,3b,4,5,6,7,7a,8a-decahydro-8H-3a,4a-epoxycyclopenta[a]-*s*-indacen-8-one	O=C(C1[C@H](N=[N+]=[N-])C(C)(C)C2)C3(C)C=C4[C@@H](N5N=NC(C6CCCC6)=C5)[C@H](C)CC47[C@](O)3[C@H](C)C12O7
**1b**	(1*S*,2*R*,3b*R*,4*S*,7*S*)-7-azido-1-(4-(cyclopentylmethyl)-1H-1,2,3-triazol-1-yl)-3b-hydroxy-2,4,6,6,8a-pentamethyl-1,2,3,3b,4,5,6,7,7a,8a-decahydro-8H-3a,4a-epoxycyclopenta[a]-*s*-indacen-8-one	O=C(C1[C@H](N=[N+]=[N-])C(C)(C)C2)C3(C)C=C4[C@@H](N5N=NC(CC6CCCC6)=C5)[C@H](C)CC47[C@](O)3[C@H](C)C12O7
**1c**	(1*S*,2*R*,3b*R*,4*S*,7*S*)-7-azido-1-(4-cyclohexyl-1H-1,2,3-triazol-1-yl)-3b-hydroxy-2,4,6,6,8a-pentamethyl-1,2,3,3b,4,5,6,7,7a,8a-decahydro-8H-3a,4a-epoxycyclopenta[a]-*s*-indacen-8-one	O=C(C1[C@H](N=[N+]=[N-])C(C)(C)C2)C3(C)C=C4[C@@H](N5N=NC(C6CCCCC6)=C5)[C@H](C)CC47[C@](O)3[C@H](C)C12O7
**1d**	(1*S*,2*R*,3b*R*,4*S*,7*S*)-7-azido-1-(4-(cyclohexylmethyl)-1H-1,2,3-triazol-1-yl)-3b-hydroxy-2,4,6,6,8a-pentamethyl-1,2,3,3b,4,5,6,7,7a,8a-decahydro-8H-3a,4a-epoxycyclopenta[a]-*s*-indacen-8-one	O=C(C1[C@H](N=[N+]=[N-])C(C)(C)C2)C3(C)C=C4[C@@H](N5N=NC(CC6CCCCC6)=C5)[C@H](C)CC47[C@](O)3[C@H](C)C12O7
**1e**	(1*S*,2*R*,3b*R*,4*S*,7*S*)-7-azido-3b-hydroxy-2,4,6,6,8a-pentamethyl-1-(4-phenyl-1H-1,2,3-triazol-1-yl)-1,2,3,3b,4,5,6,7,7a,8a-decahydro-8H-3a,4a-epoxycyclopenta[a]-*s*-indacen-8-one	O=C(C1[C@H](N=[N+]=[N-])C(C)(C)C2)C3(C)C=C4[C@@H](N5N=NC(C6=CC=CC=C6)=C5)[C@H](C)CC47[C@](O)3[C@H](C)C12O7
**1f**	(1*S*,2*R*,3b*R*,4*S*,7*S*)-7-azido-1-(4-benzyl-1H-1,2,3-triazol-1-yl)-3b-hydroxy-2,4,6,6,8a-pentamethyl-1,2,3,3b,4,5,6,7,7a,8a-decahydro-8H-3a,4a-epoxycyclopenta[a]-*s*-indacen-8-one	O=C(C1[C@H](N=[N+]=[N-])C(C)(C)C2)C3(C)C=C4[C@@H](N5N=NC(CC6=CC=CC=C6)=C5)[C@H](C)CC47[C@](O)3[C@H](C)C12O7

**2a**	(1*S*,2*R*,3b*R*,4*S*,7*S*)-1,7-bis(4-cyclopentyl-1H-1,2,3-triazol-1-yl)-3b-hydroxy-2,4,6,6,8a-pentamethyl-1,2,3,3b,4,5,6,7,7a,8a-decahydro-8H-3a,4a-epoxycyclopenta[a]-*s*-indacen-8-one	O=C(C1[C@H](N2C=C(C3CCCC3)N=N2)C(C)(C)C4)C5(C)C=C6[C@@H](N7N=NC(C8CCCC8)=C7)[C@H](C)CC69[C@](O)5[C@H](C)C14O9
**2b**	(1*S*,2*R*,3b*R*,4*S*,7*S*)-1,7-bis(4-(cyclopentylmethyl)-1H-1,2,3-triazol-1-yl)-3b-hydroxy-2,4,6,6,8a-pentamethyl-1,2,3,3b,4,5,6,7,7a,8a-decahydro-8H-3a,4a-epoxycyclopenta[a]-*s*-indacen-8-one	O=C(C1[C@H](N2C=C(CC3CCCC3)N=N2)C(C)(C)C4)C5(C)C=C6[C@@H](N7N=NC(CC8CCCC8)=C7)[C@H](C)CC69[C@](O)5[C@H](C)C14O9
**2c**	(1*S*,2*R*,3b*R*,4*S*,7*S*)-1,7-bis(4-cyclohexyl-1H-1,2,3-triazol-1-yl)-3b-hydroxy-2,4,6,6,8a-pentamethyl-1,2,3,3b,4,5,6,7,7a,8a-decahydro-8H-3a,4a-epoxycyclopenta[a]-*s*-indacen-8-one	O=C(C1[C@H](N2C=C(C3CCCCC3)N=N2)C(C)(C)C4)C5(C)C=C6[C@@H](N7N=NC(C8CCCCC8)=C7)[C@H](C)CC69[C@](O)5[C@H](C)C14O9
**2d**	(1*S*,2*R*,3b*R*,4*S*,7*S*)-1,7-bis(4-(cyclohexylmethyl)-1H-1,2,3-triazol-1-yl)-3b-hydroxy-2,4,6,6,8a-pentamethyl-1,2,3,3b,4,5,6,7,7a,8a-decahydro-8H-3a,4a-epoxycyclopenta[a]-*s*-indacen-8-one	O=C(C1[C@H](N2C=C(CC3CCCCC3)N=N2)C(C)(C)C4)C5(C)C=C6[C@@H](N7N=NC(CC8CCCCC8)=C7)[C@H](C)CC69[C@](O)5[C@H](C)C14O9
**2e**	(1*S*,2*R*,3b*R*,4*S*,7*S*)-3b-hydroxy-2,4,6,6,8a-pentamethyl-1,7-bis(4-phenyl-1H-1,2,3-triazol-1-yl)-1,2,3,3b,4,5,6,7,7a,8a-decahydro-8H-3a,4a-epoxycyclopenta[a]-*s*-indacen-8-one	O=C(C1[C@H](N2C=C(C3=CC=CC=C3)N=N2)C(C)(C)C4)C5(C)C=C6[C@@H](N7N=NC(C8=CC=CC=C8)=C7)[C@H](C)CC69[C@](O)5[C@H](C)C14O9
**2f**	(1*S*,2*R*,3b*R*,4*S*,7*S*)-1,7-bis(4-benzyl-1H-1,2,3-triazol-1-yl)-3b-hydroxy-2,4,6,6,8a-pentamethyl-1,2,3,3b,4,5,6,7,7a,8a-decahydro-8H-3a,4a-epoxycyclopenta[a]-*s*-indacen-8-one	O=C(C1[C@H](N2C=C(CC3=CC=CC=C3)N=N2)C(C)(C)C4)C5(C)C=C6[C@@H](N7N=NC(CC8=CC=CC=C8)=C7)[C@H](C)CC69[C@](O)5[C@H](C)C14O9
**2g**	(1*S*,2*R*,3b*R*,4*S*,7*S*)-3b-hydroxy-2,4,6,6,8a-pentamethyl-1,7-bis(4-phenethyl-1H-1,2,3-triazol-1-yl)-1,2,3,3b,4,5,6,7,7a,8a-decahydro-8H-3a,4a-epoxycyclopenta[a]-*s*-indacen-8-one	O=C(C1[C@H](N2C=C(CCC3=CC=CC=C3)N=N2)C(C)(C)C4)C5(C)C=C6[C@@H](N7N=NC(CCC8=CC=CC=C8)=C7)[C@H](C)CC69[C@](O)5[C@H](C)C14O9
